# KLK8/HGF/Met signaling pathway mediates diabetes-associated hippocampal neuroinflammation in male mice

**DOI:** 10.7150/thno.109513

**Published:** 2025-05-25

**Authors:** Dan-Hong Xu, Xiao-Yong Zhang, Shi-Yu Liu, Juan Wei, Jun-Hui Zhan, Jian-Kui Du, Yu-Jian Liu, Xiao-Yan Zhu

**Affiliations:** 1 Department of Physiology, Naval Medical University, Shanghai, 200433, China.; 2 School of Kinesiology, The Key Laboratory of Exercise and Health Sciences of Ministry of Education, Shanghai University of Sport, Shanghai, 200438, China.; 3 School of Sports and Health, Nanjing Sport Institute, Nanjing, 210014, China.

**Keywords:** aerobic exercise, tissue kallikrein-related peptidase 8, hepatocyte growth factor, microglia activation, depression, neuroinflammation, diabetes

## Abstract

**Rationale:** Neuroinflammation plays a critical role in the pathogenesis of diabetes-associated depression. Tissue kallikrein-related peptidase 8 (KLK8), a secreted serine protease, has been implicated in the pathogenesis of depression- and anxiety-related behaviors across various etiologies, however the underlying mechanisms remain largely unexplored. This study elucidates a novel mechanism by which KLK8 upregulation contributes to diabetes-induced microglial activation and neuroinflammation in the hippocampus through modulating the hepatocyte growth factor (HGF)/Met signaling pathway.

**Methods and Results:** Streptozotocin (STZ)-induced diabetic mice exhibited increased KLK8 expression in the hippocampus, an effect that was mitigated in KLK8-deficient or aerobic running-exercised mice. KLK8 deficiency significantly reduced depression-like behaviors, microglial activation, and neuroinflammation in diabetic mice. In BV2 mouse microglial cells, adenovirus-mediated overexpression of KLK8 (Ad-KLK8) was sufficient to induce microglial activation. Co-immunoprecipitation (Co-IP) coupled with mass spectrometry revealed that CD44 might interact with KLK8. KLK8 overexpression decreased CD44 levels in microglial cells. However, the CD44 activator Angstrom6 further exacerbated KLK8-induced microglial activation. Conversely, transcriptional profiling of KLK8-overexpressing microglial cells and subsequent validation demonstrated that the Met/Src/Btk/NF-κB signaling pathway played a central role in mediating the stimulatory effects of KLK8 on microglial activation in both Ad-KLK8-treated BV2 cells and human microglial cell line HMC3 cells stably transfected with KLK8 lentivirus (Lv-KLK8). The Met receptor is activated upon binding to its ligand HGF, which exists as an inactive precursor (pro-HGF). Our findings showed that KLK8 cleaved pro-HGF, promoting HGF release and subsequently activating the Met/Src/Btk/NF-κB signaling pathway in microglial cells. High glucose conditions increased KLK8 expression and enhanced HGF release, thereby stimulating the Met/Src/Btk/NF-κB signaling pathway and microglial activation in a KLK8-dependent manner. Systemic administration of a Met inhibitor inactivated the Met/Src/Btk/NF-κB pathway, reducing depression-like behaviors, microglial activation, and neuroinflammation in STZ-induced diabetic mice. Both Met inhibitor and KLK8 deficiency enhanced hippocampal neuroplasticity in STZ-induced diabetic mice. Finally, we demonstrated that running exercise reversed KLK8 upregulation and inactivated Met/Src/Btk/NF-κB signaling pathways, thereby attenuating neuroinflammation, improving neuroplasticity, and alleviating depression-like behaviors in STZ-induced diabetic mice.

**Conclusions:** This study provides evidence that the KLK8/HGF/Met signaling pathway mediates diabetes-associated hippocampal neuroinflammation and depression-like behaviors, highlighting the therapeutic potential of targeting this pathway in diabetes-associated depression.

## Introduction

Diabetes is a global health concern characterized by chronic hyperglycemia, leading to a variety of complications that significantly impact global health and mortality rates 1. Approximately 65% of individuals with diabetes also experience depressive symptoms, a complication that exacerbates health outcomes and diminishes quality of life 2. The precise mechanisms linking diabetes and depression remain incompletely understood. Recent studies on the pathophysiology of diabetes-associated depression have highlighted the critical role of microglia, the primary immune cells in the brain 3, 4. Comprising 5% to 10% of all brain cells, microglia play an essential role in orchestrating the inflammatory response within the central nervous system 5. Prolonged hyperglycemia can lead to excessive microglial activation, resulting in increased release of pro-inflammatory cytokines, neurological impairments, and neuronal loss 6. This neuroinflammatory pathway is increasingly recognized as a key factor in the development of depression among individuals with diabetes, underscoring the intricate interplay between metabolic and mental health disorders.

KLK8, also referred to as neuropsin, is present at high levels in the brain, particularly in regions such as the hippocampus and amygdala which are of paramount importance for regulating our emotions 7, 8. It assumes a significant role in the adaptability and learning processes of our brain, influencing our memory 7, 8. Several studies have indicated the crucial role of KLK8 in the pathogenesis of depression- and anxiety-related behaviors resulting from different etiologies 9-11. Our recent research revealed that KLK8 mediated the proteolytic processing of the NCAM1 extracellular domain, thereby exerting a pro-apoptotic effect on hippocampal neurons during the pathogenesis of chronic unpredictable mild stress (CUMS)-induced depression 11. Nevertheless, the connection between KLK8 and depression related to diabetes, particularly its role in modulating microglial cells, remains largely undetermined.

In the present study, we first examined the effects of KLK8 deficiency on hippocampal microglial activation, neuroinflammation, and depression-like behaviors in streptozotocin (STZ)-induced diabetic mice. Furthermore, we elucidated the molecular mechanisms underlying KLK8-mediated microglial activation in the context of hyperglycemia. Accumulating evidence highlights the benefits of physical exercise in alleviating depression arising from diverse causes, such as post-stroke, postpartum, and stress-related neuroendocrine disorders 12-14. Our findings further revealed that the antidepressant effects of aerobic running exercise in diabetic mice were associated with reduced KLK8 expression in the hippocampus.

## Materials and Methods

### Animals

All laboratory mice employed in this study were maintained within a pathogen-free facility at the Animal Research Center of Naval Medical University. The animal studies were conducted in accordance with the Guide for the Care and Use of Laboratory Animals published by the NIH (NIH publication No. 85-23, revised 1996), and were sanctioned by the Ethics Committee of Naval Medical University. The KLK8 knockout mice utilized in this study were generated by Shanghai Biomodel Organism Science & Technology Development Co., Ltd. (Shanghai, China) as previously described 11, 15. The KLK8-flox mouse line was developed using a LoxP targeting system, with two LoxP sites flanking exons 1-3 of the mouse KLK8 gene. Specifically, the LoxP elements were introduced into the KLK8 gene via homologous recombination in embryonic stem cells. Global KLK8 knockout mice were obtained by crossing KLK8-flox mice with EIIa-Cre transgenic mice (The Jackson Laboratory). Successful deletion of the KLK8 gene was confirmed by PCR analysis of genomic DNA using the primers (5'-GGACGTTGGAGTCACAGC-3') and (5'-CCCAGGAGCAGAAGAGTG-3'). In this study, KLK8^flox/flox^; EIIa-Cre(+) mice (KLK8^-/-^) and their age-matched KLK8^flox/flox^; EIIa-Cre(-) littermates (serving as controls) were employed to investigate the effects of KLK8 deficiency. Animal experiments were randomized using a random number table. During the measurement and analysis phases of all *in vivo* studies, both genotype and treatment were blinded. To eliminate the potential interference of different sexes, only male mice were incorporated in the experiments of this study.

### The administration of STZ

In the STZ administration protocol, diabetes was induced through intraperitoneal injection of STZ (100 mg/kg, Sigma, USA) dissolved in citrate buffer (pH: 4.5) for two consecutive days as described 16. Mice of the same age injected with an equivalent volume of citrate buffer alone served as the control group. With this protocol, approximately 90% of the mice developed hyperglycemia, as characterized by non-fasting blood glucose levels of ≥ 16.7 mmol/L on the seventh day after the final STZ injection.

### Treadmill exercise training

The treadmill exercise program was conducted as previously described 17. Initially, the mice underwent a one-week adaptation phase on the treadmill. During this period, they were trained at incrementally increasing speeds of 4, 5, 6, 8, 10, and 12 meters per minute, with each speed maintained for 5 min at a 0% slope. Following the adaptation phase, the mice in the exercise training groups engaged in regular treadmill exercise for 4 weeks, running 5 days/week at a speed of 12 m/min for 45 min per day without exhaustion. In contrast, the control and Diabetes groups remained under sedentary conditions throughout the study.

### Experimental groups and drug treatment

For the first part, the experiments were designed to investigate the impact of KLK8 deficiency on the depression-like behaviors, hippocampal microglial activation, and neuroinflammation in the STZ-induced diabetic mice. Mice were randomly assigned to the following four groups: (a) KLK8^+/+^+Control group. KLK8^+/+^ mice were intraperitoneally injected with citrate buffer. (b) KLK8^+/+^+Diabetes group. KLK8^+/+^ mice were intraperitoneally injected with STZ. (c) KLK8^-/-^+Control group. KLK8^-/-^ mice were intraperitoneally injected with citrate buffer. (d) KLK8^-/-^+Diabetes group. KLK8^-/-^ mice were intraperitoneally injected with STZ. Behavioral testing and hippocampal sample collection were conducted 5 weeks after the measurement of blood glucose levels.

For the second part, the experiments were designed to explore the impact of aerobic exercise on the depression-like behaviors, hippocampal microglial activation, and neuroinflammation in the STZ-induced diabetic mice. Mice were randomly assigned to the following four groups: (a) Sedentary+Control group. Mice were subjected to sedentary conditions and intraperitoneally injected with citrate buffer. (b) Sedentary+Diabetes group. Mice were subjected to sedentary conditions and intraperitoneally injected with STZ. (c) Exercise+Control group. Mice were subjected to regular treadmill exercise and intraperitoneally injected with citrate buffer. (d) Exercise+Diabetes group. Mice were subjected to regular treadmill exercise and intraperitoneally injected with STZ. Behavioral testing and hippocampal sample collection were conducted 5 weeks after the measurement of blood glucose levels.

For the third part, we aimed to investigate the effect of systemic administration of Met inhibitor JNJ-38877605 on the depression-like behaviors, hippocampal microglial activation, and neuroinflammation in the STZ-induced diabetic mice. JNJ-38877605 (MCE, Princeton, NJ, USA) was dissolved in corn oil. Mice were randomly allocated to the following groups: (a) Control group. Citrate buffer-treated mice were intraperitoneally injected with corn oil. (b) Diabetes group: STZ-treated mice were intraperitoneally injected with corn oil. (c) JNJ-38877605 10 mg/kg group: Citrate buffer-treated mice were intraperitoneally injected with 10 mg/kg JNJ-38877605. (d) JNJ-38877605 20 mg/kg group: Citrate buffer-treated mice were intraperitoneally injected with 20 mg/kg JNJ-38877605. (e) Diabetes+JNJ-38877605 10 mg/kg group: STZ-treated mice were intraperitoneally injected with 10 mg/kg JNJ-38877605. (f) Diabetes+JNJ-38877605 20 mg/kg group: STZ-treated mice were intraperitoneally injected with 20 mg/kg JNJ-38877605. JNJ-38877605 or corn oil treatment commenced 24 h after blood glucose measurement, administered once every two days for a period of 5 weeks. The selection of the JNJ-38877605 dosage was based on previous studies 18 and our preliminary experiments. Behavioral testing and hippocampal sample collection were conducted 5 weeks after the measurement of blood glucose levels.

Behavioral testing and sample collection were performed 5 weeks after the measurement of blood glucose levels. Mice used for behavioral tests were subsequently subjected to immunofluorescence analysis and Golgi staining (n = 7 per group). Furthermore, an additional 7 mice per group were exclusively allocated for PCR and Western blot analyses.

### Behavioral measurements

The forced swimming test (FST) was conducted in accordance with the method described previously 11 with minor modifications. Animals were placed in plexiglas cylinders (20 cm in diameter × 35 cm in height) filled with water at 23 - 25 °C. All animals were compelled to swim for 6 min, and the immobility time and swimming time were recorded during the last 5 min of the experiment.

The Tail Suspension Test (TST) was carried out as described previously 11. Each mouse was fastened with tape approximately 1 cm from the tip of the tail for 6 min, and the duration of immobility during the last 5 min of the test was recorded.

The Novelty-Suppressed Feeding Test (NSFT) was executed as described previously 11. Animals were deprived of food 24 h prior to the test. In the test, equal amounts of food were placed in the center of the apparatus (25 cm in length × 25 cm in width × 30 cm in height). The animals were placed in the corner of the apparatus, freely biting the food for 5 min, and the time to the first feeding was recorded. Immediately thereafter, the animals were returned to the cages, and food and water were provided ad libitum.

In the Open Field Test (OFT), mice were placed at the center of the open field (40 cm in length × 40 cm in width × 40 cm in height, Shanghai Ruanlong Science and Technology Development Co., Ltd., Shanghai, China) and permitted to freely explore for 5 min. A video-computerized tracking system was utilized to record the behavior of the animals. The parameters assessed were the frequency of crossing squares, the total distances traveled, and the duration of time spent in the central and peripheral areas 16. The next test was conducted after cleaning the chamber.

### Cell culture and infection of adenovirus or lentivirus

BV2 mouse microglial cell lines were purchased from Servicebio (Wuhan, China) and cultivated in Dulbecco's modified Eagle's medium (DMEM, Gibco, New York, USA) containing normal glucose (NG, 5.5 mM D-glucose),10% fetal bovine serum (FBS, Gibco, New York, USA), and 1% penicillin/streptomycin (Gibco, New York, USA) at 37 ℃ in a humidified atmosphere consisting of 95% O_2_ and 5% CO_2_. KLK8 adenovirus (Ad-KLK8) was generated by employing the AdEasyTM adenoviral vector system (Stratagene, La Jolla, CA, USA) as previously described 11. The cell infection was carried out according to the manufacturer's protocol in DMEM medium supplemented with 10% FBS.

HMC3 human microglial cell lines were obtained from Servicebio (Wuhan, China) and cultivated in DMEM (Gibco, New York, USA) containing 10% FBS (Gibco, New York, USA), and 1% penicillin/streptomycin (Gibco, New York, USA) at 37 ℃ in a humidified atmosphere consisting of 95% O_2_ and 5% CO_2_. To achieve stable expression of KLK8, HMC3 cells were transfected with KLK8-expressing lentiviral vector (Lv-KLK8), which was designed and synthesized by Shanghai GeneChem Co. (Shanghai, China). Briefly, HMC3 cells were seeded in 6-well plates at a density of 1 × 10^5^ cells/well one day prior to lentivirus infections. Then, in accordance with the reference 19 and the manufacturer's instructions, HMC3 cells were transfected with lentivirus vectors at a multiplicity of infection (MOI) of 50, which was removed and replaced with a fresh medium containing 10% FBS after 16 h of incubation. After 72 h of transfection, cells were treated with 2 μg/mL puromycin (MCE, Princeton, NJ, USA) to generate a stable KLK8-overexpression cell line. The stable overexpression efficiency of Lv-KLK8 was confirmed by qRT-PCR and western blot analysis.

### RNA Sequencing (RNA-seq) and bioinformatic analysis

BV2 for RNA-seq experiments were seeded into 6-well plates, and infected with Ad-KLK8 or Ad-Vector at a MOI of 3 in serum-free medium for 48 h. After treatment, BV2 was collected for RNA-seq analysis. Total RNA of BV2 was extracted using Trizol reagent (Vazyme, Nanjing, China). RNA-seq analysis was performed by OE Biotech (Shanghai, China). The constructed library was qualified with the Agilent 2100 Bioanalyzer, and the Illumina HiSeq™ 2500 platform was used for sequencing. The gene expression was measured in units of fragments per kilobase of exon model per million mapped reads (FPKM). A *p*-value with correction for multiple testing using the Benjamini-Hochberg (BH) procedure, BH-corrected false-discovery rate (FDR) < 0.05, and |Fold Change (FC)| ≥ 1.5 were set as the threshold for significant differential expression.

### Transfection of Small interfering RNA (siRNA)

Human and Mouse KLK8 small interfering RNA (siRNA) was synthesized by Genepharma Corp. (Shanghai, China). The target sequences for human KLK8 siRNA and mouse KLK8 siRNA are as follows: 5'-TGGAGGACCACAACCATGATCTGAT-3' and 5′-CCUGGAUCAAGAAGACCAUTT-3′, respectively. Negative control siRNA was scrambled sequence without any specific target: 5'-TTCTCCGAACGTGTCACGT-3'. Transfection of siRNA in BV2/HMC3 cells was performed by using the XfectTM RNA transfection reagent (TaKaRa, Japan) according to the manufacturer's instructions.

### Western blot and Immunoprecipitation

Hippocampus tissue, BV2 and HMC3 cells were lysed using chilled RIPA lysis buffer (Beyotime, Jiangsu, China) containing protease and phosphatase inhibitor cocktail (Beyotime, Jiangsu, China) in accordance with the manufacturer's instructions. Protein concentration was determined by BCA Protein Assay Kit (Beyotime, Jiangsu, China). Equal amounts of protein were separated by 10% SDS-PAGE and transferred to PVDF membrane (Millipore, Billerica, MA, USA). The membranes were blocked with 5% nonfat milk and then incubated with primary antibodies against KLK8 (Santa Cruz, sc-67666), p-Met (Cell Signaling Technology, 3077), Met (Cell Signaling Technology, 3127), p-Btk (Affinity Biosciences, AF8361), Btk (Cell Signaling Technology, 8547), p-p65 (Cell Signaling Technology, 3033), p65 (Cell Signaling Technology, 8242), Src (Proteintech, 11097-1-AP), Iba1 (Proteintech, 26177-1-AP), PSD-95 (Servicebio, GB11277), SYP (Servicebio, GB15553) and β-actin (Sigma-Aldrich, A5441). Subsequently, the membrane was incubated with a secondary horseradish peroxidase-conjugated antibody for 1 h at room temperature. Immunoreactive proteins were visualized using the chemiluminescence imaging system (Tanon, Shanghai, China).

For the Immunoprecipitation assay, BV2 cell lysates were incubated on ice for 2 h, and cell debris was removed by centrifugation. The clarified supernatants were incubated with antibodies against KLK8 (Santa Cruz, sc-67666) and CD44 (Proteintech, 60224-1-Ig) at 4 °C for 16 h with gentle rotation. IgG was used as a control for nonspecific interaction. Protein A and G sepharose beads (Beyotime, Jiangsu, China) were added and incubated at 4 °C for an additional 3 h, and the immune complexes were washed four times. The final precipitate was boiled in a protein loading buffer for 5 min and eluted on 10% SDS-PAGE for western blot analysis using the respective antibodies.

### Quantitative Real-Time Polymerase Chain Reaction (PCR) Analysis

Total RNA of hippocampal tissues, BV2 microglial cells and HMC3 microglial cells was extracted by Trizol Reagent (Life Technologies, California, USA) in accordance with the manufacturer's instructions. 1 μg RNA was utilized for reverse transcription to synthesize cDNAs in a 20 μL reaction system using a PrimeScriptTM RT reagent Kit (TaKaRa, Japan). The quantitative PCR for target mRNA expression was conducted with a QuantStudio 7 Real-Time PCR detection biosystem. β-actin was employed as an internal control. The quantification of relative gene expression was analyzed by the comparative threshold cycle (CT) method with formulae (2^-ΔΔCT^). The primer sequences used in the PCR reactions are listed in Supplemental Table 1 (Supplemental Table S1).

### Measurements of HGF release

The contents of HGF in cell culture supernatant were determined using commercial ELISA kits (R&D, Minneapolis, MN, USA) following the manufacturer's instruction.

### Immunofluorescence

Hippocampal cryosections (8 μm) were blocked with 5% goat serum for 2 h at room temperature and then incubated overnight at 4 °C with primary antibodies against Iba1 (Servicebio, GB15105), p-Met (Cell Signaling Technology, 3077), p-Btk (Affinity Biosciences, AF8361), p-p65 (Cell Signaling Technology, 3033) and PSD-95 (Servicebio, GB11277). After washes, the sections were incubated with the corresponding secondary antibody at 37 °C for 1 h in the dark. The sections were counterstained with DAPI for 10 min at room temperature. The fluorescent images were captured by Pannoramic MIDI (3D HISTECH, Budapest, Hungary) and analyzed using ImageJ software.

### Golgi staining

Golgi staining was performed using a kit from Servicebio (Wuhan, China) according to the manufacturer's instructions, as previously described 20. Briefly, brain tissues were rapidly excised and rinsed in double-distilled water, followed by immersion in impregnation solutions A and B. The samples were then incubated in a dark, well-ventilated room at room temperature for 14 days while shielded from light. Subsequently, the brain sections were stained with solution C following the specified protocols. Brain tissue was sectioned into 60-micron slices using an oscillating microtome and mounted on gelatin-coated slides for staining. Dendritic spines in the hippocampus were visualized under an optical microscope (Olympus, Japan) and analyzed using Fiji software.

### Mass Spectrometry

Mass Spectrometry and Bioinformatics were carried out as described previously 11. Proteins from Ad-KLK8-treated BV2 cells were immunoprecipitated with primary antibodies against KLK8. The immunoprecipitates were separated by SDS-PAGE and stained with the Colloidal Blue Staining kit (Beyotime, Jiangsu, China). Protein sections from the SDS-PAGE gel were digested in the gel with trypsin to extract the peptide markers, and the resulting peptide mixture was resuspended in 1% formic acid and identified by ultra performance liquid chromatography tandem mass spectrometry (UPLC-MS/MS) (Bioclouds, Shanghai, China). Briefly, peptide samples were separated with the nano ACQUITY UPLC (Waters Corporation, Milford) and detected with the Q Exactive hybrid quadrupole-Orbitrap mass spectrometer (Thermo Fisher Scientific, Waltham, Massachusetts, USA). The MS/MS spectra were preprocessed with PEAKS studio version 8.5 (Bioinfor Inc., CA), and the PEAKS DB was searched against the Rattus database (UniProtKB/Swiss-Prot). The following search parameters were used: Fixed modifications: Carbamidomethyl (C); Acetylation (Protein N-term), Deamidation (NQ); Variable modifications: Oxidation (M); Missed cleavages: 2; MS mass tolerance: ± 10.0 ppm; MSMS mass tolerance: ± 0.02 Da.

### Analysis of Gene Expression Omnibus datasets

The gene chip dataset and single-cell RNA sequencing (scRNAseq) datasets were retrieved from the NCBI Gene Expression Omnibus (GEO, https://www.ncbi.nlm.nih.gov/geo/). The GEO dataset GSE34451 was analyzed to identify the differentially expressed genes (DEGs) in the hippocampus obtained from the STZ-induced diabetic rat model as compared with those from control animals. Genes with a* p* value < 0.05 and the |LogFC| ≥ 1.5 were regarded as DEGs. Two published scRNAseq datasets (GSE217045, GSE201644) related to hippocampal tissues from diabetic mice were analyzed to explore the alterations of microglial KLK8 expression under high glucose conditions.

### Statistical analysis

All data are expressed as means ± SEM. Two-tailed unpaired t-tests was used to compare the differences between the means of two groups. One-way or two-way analysis of variance (ANOVA) with Bonferroni's post hoc test was performed for comparisons among multiple groups using SPSS 22.0 (SPSS Inc., Chicago, USA).* p* < 0.05 was considered statistically significant.

## Results

### KLK8 deficiency mitigates depression-like behaviors, microglia activation, and neuroinflammation in STZ-induced diabetic mice

**Figure 1:**
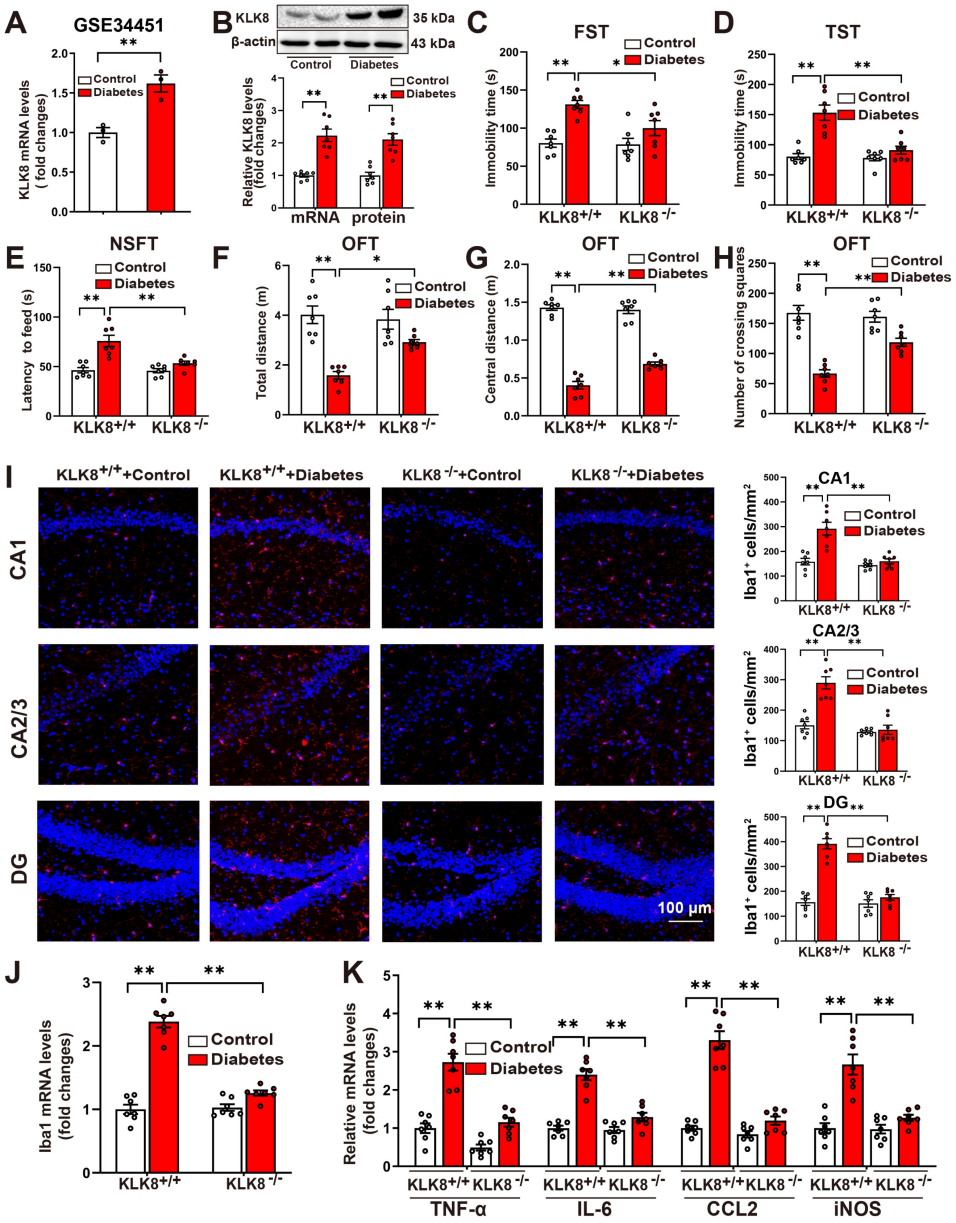
** KLK8 deficiency mitigates depressive-like behaviors, microglia activation, and neuroinflammation in STZ-induced diabetic mice. A,** Published GEO dataset GSE34451 was analyzed for hippocampal KLK8 expression in STZ-induced diabetic rats (n = 3, unpaired t-test, *p* = 0.008). **B,** The levels of KLK8 mRNA and protein were measured in the hippocampus after 5 weeks of STZ-induced diabetes by qRT-PCR (n = 7, unpaired t-test, *p* < 0.001) and western blotting (n = 7, unpaired t-test,* p* < 0.001), respectively. Representative protein bands were presented on the top of the histograms. **C-H,** Depressive behavioral tests were performed in wild-type and KLK8^-/-^ mice after 5 weeks of STZ-induced diabetes. **C,** The immobility time in the FST (n = 7, two-way ANOVA, F_1,24 (genotype)_ = 5.006, *p* = 0.035; F_1,24 (treatment)_ = 24.253, *p* < 0.001; F_1,24 (genotype×treatment)_ = 4.039, *p* = 0.056). **D,** The immobility time in the TST (n = 7, two-way ANOVA, F_1,24 (genotype)_ = 16.824, *p* < 0.001; F_1,24 (treatment)_ = 29.507, *p* < 0.001; F_1,24 (genotype×treatment)_ = 14.443, *p* < 0.001). **E,** The latency to feed in the NSFT (n = 7, two-way ANOVA, F_1,24 (genotype)_ = 10.934, *p* = 0.003; F_1,24 (treatment)_ = 28.872, *p* < 0.001; F_1,24 (genotype×treatment)_ = 10.129, *p* = 0.004). **F,** Total distance in the OFT (n = 7, two-way ANOVA, F_1,24 (genotype)_ = 4.171, *p* = 0.052; F_1,24 (treatment)_ = 35.471, *p* < 0.001;F_1,24 (genotype×treatment)_ = 7.207, *p* = 0.013). **G,** Central distance in the OFT (n = 7, two-way ANOVA, F_1,24 (genotype)_ = 9.261, *p* = 0.006; F_1,24 (treatment)_ = 438.822, *p* < 0.001;F_1,24 (genotype×treatment)_ = 14.077, *p* < 0.001). **H,** Number of crossing squares in the OFT (n = 7, two-way ANOVA, F_1,24 (genotype)_ = 6.572, *p* = 0.017; F_1,24 (treatment)_ = 65.507, *p* < 0.001; F_1,24 (genotype×treatment)_ = 10.818, *p* = 0.003). **I,** Immunofluorescent staining showed Iba1 (red) expression in the CA1, CA2/3, and DG subregions of the hippocampus in wild-type and KLK8^-/-^ mice after 5 weeks of STZ-induced diabetes. Nuclei were counterstained with DAPI (blue). Scale bar = 100 μm. The quantifications of Iba1^+^ cell numbers in each subfield of the hippocampus were presented in the right panels (n = 7, two-way ANOVA, CA1: F_1,24 (genotype)_ = 21.571, *p* < 0.001; F_1,24 (treatment)_ = 22.334,* p* < 0.001; F_1,24 (genotype×treatment)_ = 13.913, *p* = 0.001. CA2/3: F_1,24 (genotype)_ = 39.5, *p* < 0.001; F_1,24 (treatment)_ = 27.288, *p* < 0.001; F_1,24 (genotype×treatment)_ = 22.219, *p* < 0.001. DG: F_1,24 (genotype)_ = 51.154, *p* < 0.001; F_1,24 (treatment)_ = 70.554, *p* < 0.001; F_1,24 (genotype×treatment)_ = 46.632, *p* < 0.001). **J,** The mRNA expression levels of Iba1 in the hippocampus of wild-type and KLK8^-/-^ mice after 5 weeks of STZ-induced diabetes were detected by qRT-PCR (n = 7, two-way ANOVA, F_1,24 (genotype)_ = 65.845, *p* < 0.001; F_1,24 (treatment)_ = 141.79, *p* < 0.001; F_1,24 (genotype×treatment)_ = 72.842, *p* < 0.001). **K,** The mRNA expression levels of TNF-α, IL-6, CCL2, and iNOS in the hippocampus of wild-type and KLK8^-/-^ mice after 5 weeks of STZ-induced diabetes were detected by qRT-PCR (n = 7, two-way ANOVA, TNF-α: F_1,24 (genotype)_ = 50.468, *p* < 0.001; F_1,24 (treatment)_ = 66.765, *p* < 0.001; F_1,24 (genotype×treatment)_ = 13.231, *p* = 0.001. IL-6: F_1,24 (genotype)_ = 30.734, *p* < 0.001; F_1,24 (treatment)_ = 69.409, *p* < 0.001; F_1,24 (genotype×treatment)_ = 25.936, *p* < 0.001. CCL2: F_1,24 (genotype)_ = 65.209, *p* < 0.001; F_1,24 (treatment)_ = 90.244, *p* < 0.001; F_1,24 (genotype×treatment)_ = 48.218, *p* < 0.001. iNOS: F_1,24 (genotype)_ = 19.097, *p* < 0.001; F_1,24 (treatment)_ = 36.559, *p* < 0.001; F_1,24 (genotype×treatment)_ = 17.733, *p* < 0.001). Data were presented as means ± SEM. * *p* < 0.05, ** *p* < 0.01.

To explore the mechanisms underlying the diabetes-induced microglial activation and neuroinflammation in the hippocampus, we analyzed a published GEO dataset (GSE34451) and identified 1183 upregulated DEGs and 203 downregulated DEGs in the hippocampus obtained from the STZ-induced diabetic rat model compared with those from control animals (Supplemental Table S2). Among these DEGs, KLK8, a gene implicated in the pathogenesis of stress-induced depression-like behaviors 7, 8, 11, was significantly upregulated in the hippocampus obtained from the STZ-induced diabetes group compared with those from the control group (Figure 1A). Validation through qRT-PCR and western blotting confirmed the upregulation of KLK8 in the hippocampus of STZ-induced diabetic mice (Figure 1B).

Subsequently, we examined whether KLK8 deficiency influenced the depression-like behaviors in STZ-induced diabetic mice. As anticipated, the upregulation of hippocampal KLK8 induced by diabetes was blunted in KLK8-deficient (KLK8^-/-^) mice (Supplemental Figure S1A). Additionally, STZ-induced diabetic mice had significantly longer immobility times in the FST (Figure 1C) and the TST (Figure 1D), as well as an increase in the latency to feed in the NSFT (Figure 1E), compared to control mice. Anxiety is a common symptom of depression. OFT were then employed to assess anxiety-like behavior in mice. STZ-induced diabetic mice displayed significant decreases in the total distance traveled in the test field (Figure 1F), in the distance traveled in the central area of the test field (Figure 1G), and the number of crossing squares (Figure 1H), when compared to control mice. These results confirmed the emergence of depression-like behavior in STZ-induced diabetic mice.

Notably, the STZ-induced increases in the immobility time in the FST (Figure 1C) and the TST (Figure 1D), as well as the increases in the latency to feed in the NSFT (Figure 1E), were dramatically decreased in KLK8^-/-^ mice. Moreover, KLK8 deficiency significantly increased the distance traveled in the test field (Figure 1F), the distance traveled in the central area of the test field (Figure 1G), and the number of crossing squares (Figure 1H) in the OFT in STZ-induced diabetic mice. These results indicated that KLK8 deficiency alleviated STZ-induced depression-like behaviors.

Microglial activation and neuroinflammation are important contributors to depression pathogenesis. We then investigated the effect of KLK8 deficiency on the number of microglia by quantitatively analyzing the number of Iba1^+^ cells in three subregions of the hippocampus through immunofluorescence staining. The results revealed that the numbers of Iba1^+^ cells in the Cornus Ammonis (CA)1, CA2/3, and dentate gyrus (DG) subregions of the hippocampus were significantly increased in the STZ-induced diabetic mice compared to the control mice (Figure 1I). However, KLK8 deficiency reduced Iba1^+^ cells in each subfield of the hippocampus in the STZ-induced diabetic mice (Figure 1I). Moreover, compared with the control mice, the STZ-induced diabetic mice had significantly higher Iba1 mRNA levels, which were decreased by KLK8 deficiency (Figure 1J).

Microglia represent the primary immune cell type in the hippocampus to orchestrate a potent pro-inflammatory response during the pathogenesis of depression. qRT-PCR analysis demonstrated that the expression of proinflammatory cytokines and chemokines, including tumor necrosis factor-α (TNF-α), interleukin-6 (IL-6), CC Chemokine Ligand 2 (CCL2), as well as induced nitric oxide synthase (iNOS), in the hippocampus was increased in the STZ-induced diabetic mice compared to the control mice (Figure 1K). However, KLK8 deficiency also markedly reduced the STZ-induced mRNA expression of proinflammatory cytokines, chemokines, and iNOS in the hippocampus (Figure 1K).

Taken together, these findings indicated that KLK8 deficiency attenuates depression-like behaviors, microglia activation, and neuroinflammation in STZ-induced diabetic mice.

### KLK8 promotes microglial activation via a Met-dependent signaling pathway

**Figure 2:**
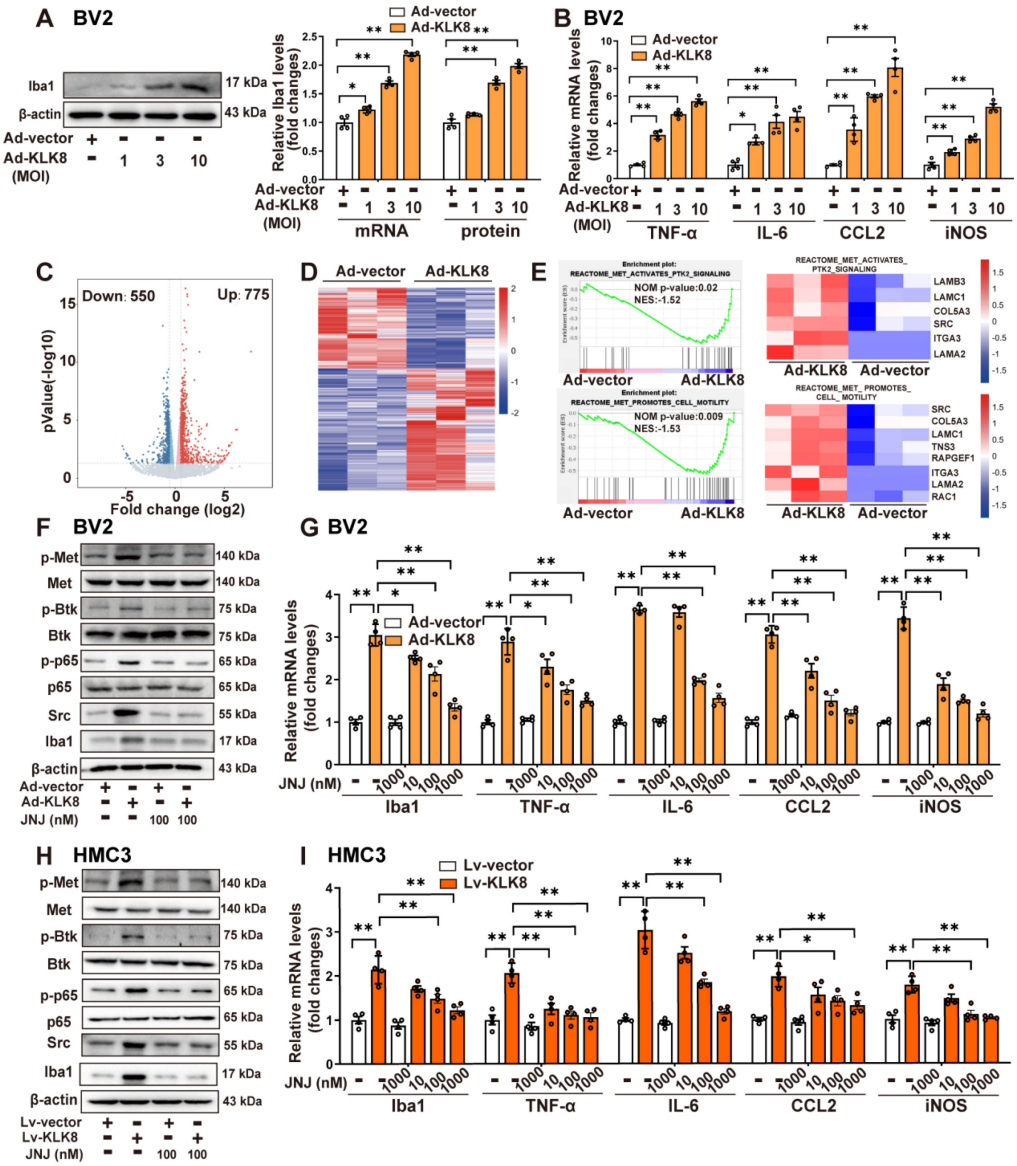
** KLK8 promotes microglial activation via a Met-dependent signaling pathway. A-B**, BV2 mouse microglial cells were infected with Ad-KLK8 at a MOI of 1, 3, or 10 for 48 h.** A**, mRNA and protein expression levels of Iba1 were detected by qRT-PCR (F_3,12_ = 167.971, *p* < 0.001) and western blotting (F_3,12_ = 124.428, *p* < 0.001), respectively. Representative protein bands were presented on the left of the histograms. **B**, The mRNA expression levels of TNF-α, IL-6, CCL2, and iNOS were detected by qRT-PCR (TNF-α: F_3,12_ = 225.322, *p* < 0.001. IL-6: F_3,12_ = 23.992, *p* < 0.001. CCL2: F_3,12_ = 59.73, *p* < 0.001. iNOS: F_3,12_ = 127.568, *p* < 0.001). **C-E**, BV2 cells were infected with Ad-Vector or Ad-KLK8 at a MOI of 3 for 48 h. Dysregulated genes were analyzed by RNA-seq.** C**, Volcano plots showing DEGs in Ad-KLK8-treated BV2 cells. Red reflects upregulated and blue indicates downregulated genes. **D,** Heat map of DEGs, red indicates upregulated and blue shows downregulated genes. **E**, GSEA was then performed to determine the enriched signaling pathways in KLK8-overexpressed BV2 cells. GSEA gene sets associated with Met-activates-PTK2 (top) and Met-promotes-cell-motility (bottom) signaling pathways were significantly enriched in the Ad-KLK8-treated BV2 cells. Heat maps of the dysregulated target genes of Met-activates-PTK2 and Met-promotes-cell-motility (bottom) signaling pathways in Ad-KLK8 treated BV2 were presented on the right of the GSEA plots. Red reflects upregulated and blue indicates downregulated genes. **F-G**, BV2 cells were infected with Ad-KLK8 at a MOI of 3 for 48 h in the presence or absence of the Met inhibitor JNJ-38877605 at the indicated concentrations. **H-I,** A stably KLK8-overexpressing HMC3 cell line was generated through infection with Lv-KLK8. Cells infected with an empty lentivirus served as the control group (Lv-Vector). Stably KLK8-overexpressing HMC3 cells and control cells were treated with or without the Met inhibitor JNJ-38877605 at the indicated concentrations for 48 h. Protein levels of p-Met, Met, p-Btk, Btk, p-p65, p65, Src, and Iba1 in BV2 cells **(F)** or HMC3 cells** (H)** were determined by western blot analysis**.** The mRNA expression levels of Iba1, TNF-α, IL-6, CCL2, and iNOS in BV2 cells** (G,** Iba1: F_5,18_ = 68.758, *p* < 0.001. TNF-α: F_5,18_ = 43.501, *p* < 0.001. IL-6: F_5,18_ = 259.947, *p* < 0.001. CCL2: F_5,18_ = 61.644, *p* < 0.001. iNOS: F_5,18_ = 111.889, *p* < 0.001) or HMC3 cells** (I,** Iba1: F_5,18_ = 23.051, *p* < 0.001. TNF-α: F_5,18_ = 17.202, *p* < 0.001. IL-6: F_5,18_ = 61.04, *p* < 0.001. CCL2: F_5,18_ = 12.358, *p* < 0.001. iNOS: F_5,18_ = 19.215, *p* < 0.001**)** were detected by qRT-PCR**.** Data were presented as means ± SEM (n = 4, one-way ANOVA). * *p* < 0.05, ** *p* < 0.01. JNJ represents JNJ-38877605.

The present study subsequently confirmed the effect of KLK8 on microglial cells *in vitro*. Infection of BV2 mouse microglial cells with increasing concentrations of KLK8 adenovirus (Ad-KLK8) resulted in a corresponding augmentation in the mRNA and protein expression levels of KLK8 (Supplemental Figure S1B) and Iba1 (Figure 2A). Additionally, Ad-KLK8 treatment for 48 h dose-dependently elevated the mRNA expression levels of proinflammatory cytokines and chemokines, including TNF-α, IL-6, and CCL2, as well as iNOS, in BV2 cells (Figure 2B). These findings imply that the overexpression of KLK8 alone can instigate the activation of microglia.

As a secreted serine protease, KLK8 is known to cleave the extracellular portion of several membrane proteins, including neuregulin-1, synaptic adhesion molecule L1, and NCAM1 9, 11, 21, 22. Using co-immunoprecipitation (Co-IP) combined with mass spectrometry, the present study initially identified proteins associated with KLK8 in BV2 microglial cells treated with Ad-KLK8. Mass spectrometry disclosed that CD44, a membrane receptor known to modulate microglial activation 23, 24, was the potential matched protein (Supplemental Table S3). As shown in Supplemental Figure S2A, CD44 was co-immunoprecipitated by anti-KLK8, and vice versa, in BV2 cells. In addition, western blot analysis affirmed that KLK8 overexpression led to the downregulation of CD44 in BV2 cells (Supplemental Figure S2B). Unexpectedly, we discovered that the CD44 activator Angstrom6 per se promoted the activation of microglia and neuroinflammation, and further exacerbated the activation of microglia and the expression of inflammatory factors induced by Ad-KLK8 (Supplemental Figure S2C). Therefore, we surmise that the downregulation of CD44 is not the cause of microglial activation and the increased expression of inflammatory factors in microglial cells treated with Ad-KLK8.

To further elucidate the mechanisms underlying KLK8-induced microglial activation, we subsequently performed RNA-seq analysis of BV2 cells treated with Ad-KLK8 or Ad-Vector (n = 3 per group). We identified 775 up-regulated and 550 down-regulated DEGs in Ad-KLK8-treated BV2 cells compared with those in Ad-Vector-treated BV2 cells (|FC| ≥ 1.5, false discovery rate < 0.05, Figure 2C-D). Gene set enrichment analysis (GSEA) was then conducted to determine the enriched biological pathways in KLK8-overexpressing BV2 cells. By using Hallmark, Kyoto Encyclopedia of Genes and Genomes (KEGG), Gene Ontology (GO), and Reactome pathway analyses of GSEA, gene sets associated with Inflammatory response, TNFA signaling via NFκB, IL6-JAK-STAT3 signaling, and Met signaling pathway were significantly enriched in Ad-KLK8-treated BV2 cells (Figure 2E, Supplemental Figure S3A-C).

Met activation leads to the recruitment of intracellular effector molecules such as Src, thereby activating downstream signaling pathways and regulating inflammatory responses 25, 26. Recently, it was also discovered that Met contributes to the pathogenesis of brain injury by activating the Btk/NF-κB pathway in microglia 27. We found that Ad-KLK8 treatment led to significant increments in the phosphorylation of Met, Btk, and NF-κB p65 subunit, as well as Src expression, which were blocked by the Met inhibitor JNJ-38877605 in BV2 microglial cells (Figure 2F, Supplemental Figure S3D). Additionally, the treatment of BV2 cells with increasing concentrations of JNJ-38877605 (10~1000 nM) dose-dependently inhibited Ad-KLK8-induced BV2 microglial activation, as evidenced by the downregulation of Iba1 and the decreased mRNA expression levels of TNF-α, IL-6, CCL2, and iNOS (Figure 2G).

We then established Lv-KLK8-mediated stable KLK8 overexpression in the human microglial cell line HMC3 cells to confirm the role of the Met signaling pathway in KLK8-induced microglial activation. The efficacy of KLK8 overexpression in HMC3 microglial cells was confirmed by qRT-PCR and western blot analysis (Supplemental Figure S1C). As shown in Figure 2H and Supplemental Figure S3E, it was found that the Met inhibitor JNJ-38877605 also inhibited the stable KLK8 overexpression-induced activation of the Met/Src/Btk/NF-κB signaling pathway in HMC3 cells. The inhibitory effects of the Met inhibitor JNJ-38877605 on Lv-KLK8-induced HMC3 microglial activation also mirrored the results observed in Ad-KLK8-treated BV2 microglial cells (Figure 2I). These results suggest that KLK8 may promote microglial activation through a Met-dependent signaling pathway.

### KLK8 cleaves pro-HGF and augments HGF release, thereby facilitating Met signaling and microglial activation

We then delved into the mechanisms by which KLK8 initiates the activation of the Met signaling pathway. Conventionally, the Met receptor is activated through binding with its ligand HGF, which leads to its homo-dimerization and subsequently triggers the downstream signaling pathways 28. HGF naturally exists as an inactive precursor, known as pro-HGF. For it to fulfill its vital biological functions, pro-HGF must be transformed into its mature, active form via a process of proteolytic cleavage 29. Members of the KLK family, including KLK4, KLK5, and KLK14, are acknowledged for their effective role in activating pro-HGF and converting it into its active form 30, 31. We then examined whether KLK8 mediated the proteolytic process of pro-HGF into mature HGF.

**Figure 3:**
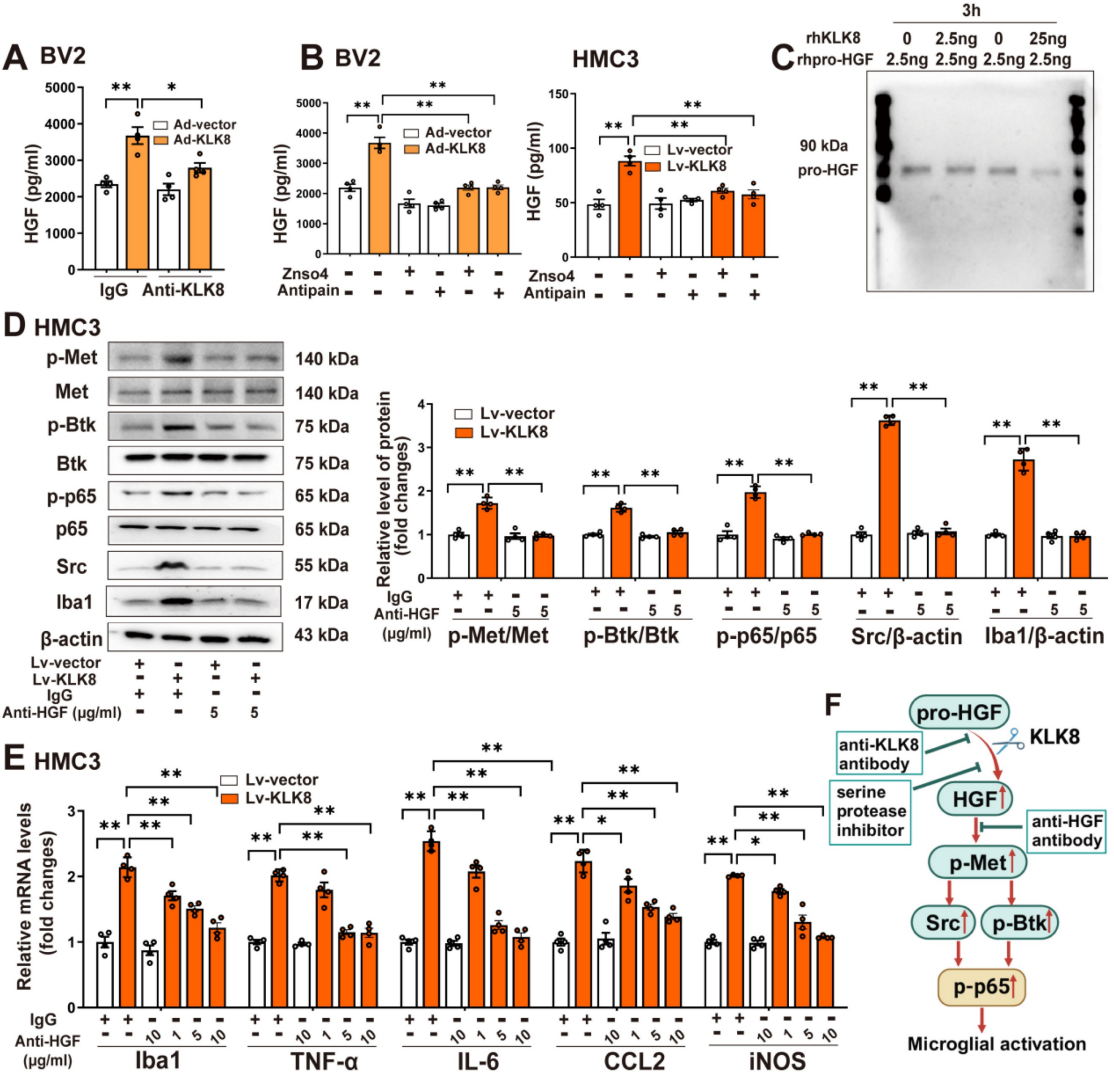
** KLK8 cleaves pro-HGF and augments HGF release, thereby facilitating Met signaling and microglial activation. A,** BV2 cells were infected with Ad-KLK8 at a MOI of 3 for 48 h with or without anti-KLK8 neutralizing antibody (2.5 μg/mL). HGF contents in the cell medium were measured by ELISA assay (F_3,12_ = 16.994, *p* < 0.001). **B,** BV2 cells were infected with Ad-KLK8 at a MOI of 3 for 48 h with or without serine protease inhibitors ZnSO4 (0.05 mM) and antipain (0.05 mM), respectively (left, F_5,18_ = 39.434, *p* < 0.001). A stably KLK8-overexpressing HMC3 cell line was generated through infection with Lv-KLK8. Cells infected with an empty lentivirus served as the Lv-Vector. Stably KLK8-overexpressing HMC3 cells and control cells were treated with or without serine protease inhibitors ZnSO4 (0.05 mM) and antipain (0.05 mM), respectively (right, F_5,18_ = 14.914, *p* < 0.001). HGF contents in the cell medium were measured by ELISA assay. **C**, Purified recombinant human pro-HGF (rhpro-HGF) was incubated with or without activated recombinant human KLK8 (rhKLK8) at the indicated concentrations, and analyzed by western blot using a pro-HGF antibody. **D-E**, A stably KLK8-overexpressing HMC3 cell line was generated through infection with Lv-KLK8. Cells infected with an empty lentivirus served as the Lv-Vector. Stably KLK8-overexpressing HMC3 cells and control cells were treated with or without a fully human anti-HGF neutralizing antibody Rilotumumab at the indicated concentrations. **D**, Protein levels of p-Met, Met, p-Btk, Btk, p-p65, p65, Src, and Iba1 were determined by western blot analysis. Representative protein bands were presented on the left of the histograms (p-Met/Met: F_3,12_ = 50.48, *p* < 0.001. p-Btk/Btk: F_3,12_ = 90.277, *p* < 0.001. p-p65/p65: F_3,12_ = 89.791, *p* < 0.001. Src/β-actin: F_3,12_ = 555.454, *p* < 0.001. Iba1/β-actin: F_3,12_ = 141.437, *p* < 0.001). **E,** The mRNA levels of Iba1, TNF-α, IL-6, CCL2, and iNOS were detected by qRT-PCR **(**Iba1: F_5,18_ = 44.23, *p* < 0.001. TNF-α: F_5,18_ = 52.255, *p* < 0.001. IL-6: F_5,18_ = 99.978, *p* < 0.001. CCL2: F_5,18_ = 41.607, *p* < 0.001. iNOS: F_5,18_ = 77.973, *p* < 0.001). **F**, Schematic diagram of the mechanism underlying KLK8-induced microglial activation. KLK8 cleaves pro-HGF and augments HGF release, leading to the activation of the Met/Src/BTK/NF-κB signaling pathway and subsequent microglial activation. Data were presented as means ± SEM (n = 4, one-way ANOVA). * *p* < 0.05, ** *p* < 0.01.

ELISA analysis disclosed that Ad-KLK8 treatment significantly enhanced the release of HGF in the culture medium of BV2 cells, and this was inhibited by the anti-KLK8 neutralizing antibody (Figure 3A). We then employed two serine protease inhibitors, Antipain and ZnSO4, to impede the proteolytic activity of KLK8 32. As depicted in Figure 3B, both Antipain and ZnSO4 effectively suppressed Ad-KLK8- or Lv-KLK8-induced HGF release in the culture medium of BV2 or HMC3 microglial cells, respectively. To determine whether KLK8 could directly cleave pro-HGF, we subsequently incubated recombinant human pro-HGF with recombinant human KLK8 at 37 °C for 3 h and observed a dose-dependent reduction in pro-HGF (Figure 3C). These findings imply that KLK8 could directly cleave pro-HGF through its proteolytic activity, thereby promoting the release of HGF.

We next observed the effect of rilotumumab, a fully human monoclonal antibody against HGF, on KLK8-induced Met signaling and microglial activation. We discovered that Lv-KLK8 treatment resulted in significant increments in phosphorylation of Met, Btk, and NF-κB p65 subunit, as well as Src expression, which were blocked by rilotumumab in HMC3 human microglial cells (Figure 3D). Additionally, treatment of HMC3 cells with increasing concentrations of rilotumumab (1~10 μg/mL) dose-dependently inhibited Lv-KLK8-induced HMC3 microglial activation, as evidenced by the downregulation of Iba1 and decreased mRNA expression levels of TNF-α, IL-6, CCL2, and iNOS (Figure 3E). These results suggest that KLK8 may promote microglial activation through a Met-dependent signaling pathway (Figure 3F).

### High glucose enhances the release of HGF, thereby stimulating Met signaling and microglial activation via a mechanism dependent on KLK8

**Figure 4:**
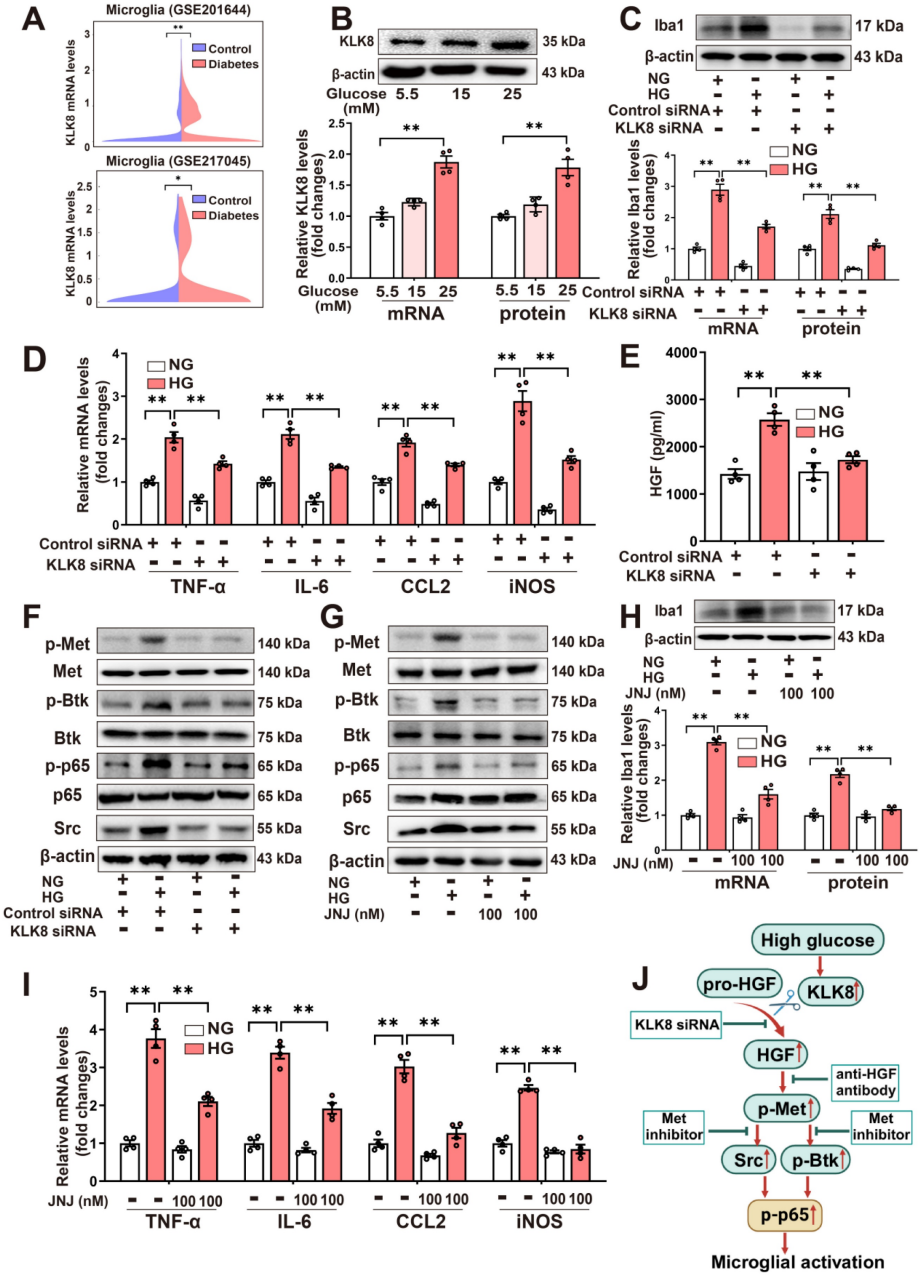
** High glucose enhances the release of HGF, thereby stimulating Met signaling and microglial activation via a mechanism dependent on KLK8. A,** Two published scRNA-seq datasets were analyzed for KLK8 mRNA expression in microglial cells from the hippocampus of db/db mice (GSE201644) and whole brain of high-fat diet-treated mice (GSE217045).** B,** BV2 cells were treated with increasing concentration of glucose (15 and 25 mM) for 48 h. The mRNA and protein expression levels of KLK8 were examined by qRT-PCR (F_2,9_ = 45.421, *p* < 0.001) and western blotting (F_2,9_ = 23.053, *p* < 0.001), respectively. Representative protein bands were presented on the top of the histograms. **C-F,** BV2 cells were transfected with control siRNA or KLK8 siRNAs, and then treated with normal glucose (NG, 5.5 mM D-glucose) or high glucose (HG, 25 mM D-glucose) for 48 h. **G-I,** BV2 cells were treated with NG or HG with or without JNJ-38877605 at the indicated concentrations for 48 h. **C** and **H,** The mRNA and protein levels of Iba1 were examined by qRT-PCR(C, F_3,12_ = 106.815, *p* < 0.001; H, F_3,12_ = 78.039, *p* < 0.001) and western blotting (C,F_3,12_ = 121.672, *p* < 0.001; H, F_3,12_ = 74.815, *p* < 0.001), respectively. Representative protein bands were presented on the top of the histograms. **D** and** I,** The mRNA levels of TNF-α, IL-6, CCL2, and iNOS were detected by qRT-PCR (D, TNF-α: F_3,12_ = 62.381, *p* < 0.001. IL-6: F_3,12_ = 78.392, *p* < 0.001. CCL2: F_3,12_ = 88.773, *p* < 0.001. iNOS: F_3,12_ = 87.725, *p* < 0.001; I, TNF-α: F_3,12_ = 81.468, *p* < 0.001. IL-6: F_3,12_ = 96.25, *p* < 0.001. CCL2: F_3,12_ = 71.734, *p* < 0.001. iNOS: F_3,12_ = 93.927, *p* < 0.001). **E,** HGF contents in the cell medium were measured by ELISA assay (F_3,12_ = 17.209, *p* < 0.001). **F** and **G,** The protein levels of p-Met, Met, p-Btk, Btk, p-p65, p65 and Src were determined by western blot analysis. **J,** Schematic diagram of the mechanism underlying high glucose-induced microglial activation. Exposure to high glucose results in the upregulation of KLK8, which subsequently enhances HGF release in microglial cells, leading to the activation of the Met/Src/BTK/NF-κB signaling pathway and subsequent microglial activation. Data were presented as means ± SEM (n = 4, one-way ANOVA). ** *p* < 0.01. JNJ represents JNJ-38877605.

To explore the alterations of microglial KLK8 expression under high glucose conditions, we initially analyzed two published single-cell RNA sequencing (scRNAseq) datasets (GSE201644, GSE217045) involving hippocampal tissues from diabetic mice. A significant upward tendency of microglia KLK8 expression was identified in the hippocampus obtained from the diabetic mice compared with those from control animals (Figure 4A, Supplemental Figure S4A-K). Validation through qRT-PCR and western blotting confirmed that BV2 microglial cells treated with escalating concentrations of glucose (15 and 25 mM) for 2 days displayed increments in the mRNA and protein expression levels of KLK8 in a dose-dependent manner (Figure 4B).

The present study next explored whether the upregulation of KLK8 contributes to high glucose-induced microglial activation using an *in vitro* model. KLK8 siRNA not only led to a significant reduction of KLK8 mRNA and protein expression in BV2 cells but also blocked the high glucose-induced upregulation of KLK8 (Supplemental Figure S1D). KLK8 knockdown significantly attenuated high glucose-induced the mRNA and protein expression levels of Iba1 (Figure 4C). Furthermore, the high glucose-induced increases in the mRNA expression levels of proinflammatory cytokines and chemokine including TNF-α, IL-6, and CCL2, as well as iNOS, were largely prevented by KLK8 knockdown in BV2 cells (Figure 4D). Collectively, these findings suggest that the upregulation of KLK8 contributes to high glucose-induced microglia activation.

As depicted in Figure 4E, we observed that high glucose (25 mM) treatment resulted in an increase in HGF release in the cell culture supernatants of the BV2 microglial cells, which was reversed by KLK8 knockdown. Additionally, we discovered that high glucose treatment also led to significant increments in phosphorylation of Met, Btk, and NF-κB p65 subunit, as well as Src expression. We found that both the KLK8 knockdown (Figure 4F, Supplemental Figure S5A) and Met inhibitor JNJ-38877605 (Figure 4G, Supplemental Figure S5B) largely inhibited the high glucose-induced activation of the Met/Src/Btk/NF-κB signaling pathways in microglial cells. Moreover, JNJ-38877605 treatment significantly attenuated the high glucose-induced BV2 microglial activation, as evidenced by the downregulation of Iba1 (Figure 4H) and decreased mRNA expression levels of TNF-α, IL-6, CCL2, and iNOS (Figure 4I).

In human HMC3 microglial cells, we found that high glucose treatment also led to an increase in HGF release, which was markedly decreased by KLK8 siRNA (Supplemental Figure S5C). Furthermore, treatment with the human anti-HGF neutralizing antibody rilotumumab (5 μg/mL) significantly inhibited the high glucose-induced activation of the Met/Src/Btk/NF-κB signaling pathway (Supplemental Figure S5D). High glucose-induced microglial activation and neuroinflammation were also largely prevented by rilotumumab treatment in HMC3 cells (Supplemental Figure S5E).

Taken together, these findings suggest that high glucose can enhance the release of HGF, thereby stimulating Met signaling and microglial activation through a mechanism dependent on KLK8 (Figure 4J).

### Met inhibitor inactivates Src/Btk/NF-κB signaling pathways and attenuates depression-like behaviors, microglia activation, and neuroinflammation in STZ-induced diabetic mice

**Figure 5:**
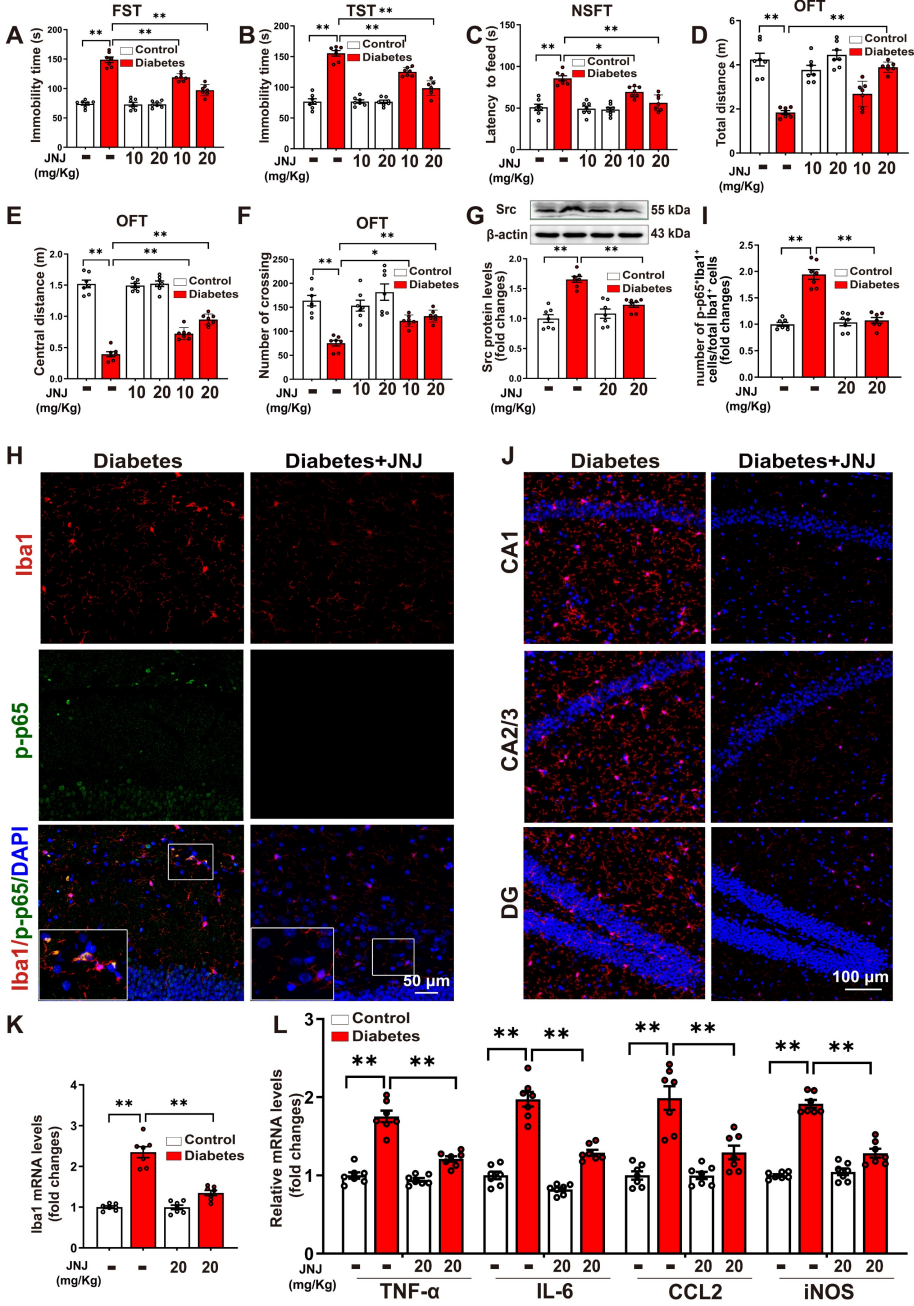
** Met inhibitor inactivates Src/Btk/NF-κB signaling pathways and attenuates depressive-like behaviors, microglia activation, and neuroinflammation in STZ-induced diabetic mice.** Control or STZ-induced diabetic mice were intraperitoneally injected with the Met inhibitor JNJ-38877605 at the indicated concentrations once every two days for a period of 5 weeks. **A-F,** Depressive behavioral tests were performed in diabetic mice injected with or without JNJ-38877605. **A,** The immobility time in the FST (F_2,36 (Met inhibitor)_ = 33.722, *p* < 0.001; F_1,36 (treatment)_ = 338.557, *p* < 0.001; F_2,36 (Met inhibitor×treatment)_ = 31.517, *p* < 0.001). **B,** The immobility time in the TST (F_2,36 (Met inhibitor)_ = 30.134, *p* < 0.001; F_1,36 (treatment)_ = 274.868, *p* < 0.001; F_2,36 (Met inhibitor×treatment)_ = 29.59, *p* < 0.001). **C,** The latency to feed in the NSFT (F_2,36 (Met inhibitor)_ = 13.162, *p* < 0.001; F_1,36 (treatment)_ = 67.763, *p* < 0.001; F_2,36 (Met inhibitor×treatment)_ = 9.069, *p* < 0.001). **D,** Total distance in the OFT (F_2,36 (Met inhibitor)_ = 18.756, *p* < 0.001; F_1,36 (treatment)_ = 69.048, *p* < 0.001; F_2,36 (Met inhibitor×treatment)_ = 11.387, *p* < 0.001). **E,** Central distance in the OFT (F_2,36 (Met inhibitor)_ = 21.682, *p* < 0.001; F_1,36 (treatment)_ = 564.581, *p* < 0.001; F_2,36 (Met inhibitor×treatment)_ = 21.773, *p* < 0.001). **F,** Number of crossing squares in the OFT (F_2,36 (Met inhibitor)_ = 6.708, *p* < 0.001; F_1,36 (treatment)_ = 47.193, *p* < 0.001; F_2,36 (Met inhibitor×treatment)_ = 4.162, *p* < 0.001). **G,** The protein levels of Src were examined by western blotting in hippocampal tissue. Representative protein bands were presented on the top of the histograms (F_1,24 (Met inhibitor)_ = 8.067, *p* < 0.001; F_1,24 (treatment)_ = 44.705, *p* < 0.001; F_1,24 (Met inhibitor×treatment)_ = 17.681, *p* < 0.001). **H,** Hippocampal sections were stained with fluorophore-labeled antibodies against the microglial cell marker Iba1 (red) and phosphorylated p65 (p-p65, green). DAPI staining was used to detect nuclei (blue). The merge image represents double positive staining for Iba1 and p-p65. Areas in white boxes were shown enlarged. Scale bar = 50 μm. **I,** The quantification of the percentage of Iba1^+^/p-p65^+^ cells in total Iba1^+^ cells (F_1,24 (Met inhibitor)_ = 41.553, *p* < 0.001; F_1,24 (treatment)_ = 57.86, *p* < 0.001; F_1,24 (Met inhibitor×treatment)_ =48.965, *p* < 0.001). J, Immunofluorescent staining showed Iba1 (red) expression in the CA1, CA2/3, and DG subregions of the hippocampus. Nuclei were counterstained with DAPI (blue). Scale bar = 100 μm. **K,** The mRNA levels of Iba1 in the hippocampus were detected by qRT-PCR (F_1,24 (Met inhibitor)_ = 39.264, *p* < 0.001; F_1,24 (treatment)_ = 112.522, *p* < 0.001; F_1,24 (Met inhibitor×treatment)_ =38.966, *p* < 0.001). **L,** The mRNA levels of TNF-α, IL-6, CCL2, and iNOS in the hippocampus were detected by qRT-PCR (TNF-α: F_1,24 (Met inhibitor)_ = 40.845, *p* < 0.001; F_1,24 (treatment)_ = 118.166, *p* < 0.001; F_1,24 (Met inhibitor)_ = 26.897, *p* < 0.001. IL-6: F_1,24 (Met inhibitor)_ =57.168, *p* < 0.001; F_1,24 (treatment)_ = 159.94, *p* < 0.001; F_1,24 (Met inhibitor×treatment)_ = 19.772, *p* < 0.001. CCL2: F_1,24 (Met inhibitor)_ = 13.547, *p* = 0.001; F_1,24 (treatment)_ = 46.115, *p* < 0.001; F_1,24 (Met inhibitor×treatment)_ = 13.512, *p* = 0.001. iNOS: F_1,24 (Met inhibitor)_ = 44.11, *p* < 0.001; F_1,24 (treatment)_ = 171.468, *p* < 0.001; F_1,24 (Met inhibitor×treatment)_ = 58.915, *p* < 0.001). Data were presented as means ± SEM (n = 7, two-way ANOVA). ** *p* < 0.01. JNJ represents JNJ-38877605.

To evaluate the anti-depression effects of the Met inhibitor, STZ-induced diabetic mice were treated with Met inhibitor JNJ-38877605 at a dose of 10 or 20 mg/kg for five weeks (Supplemental Figure S6A). It was found that the STZ-induced increases in immobility time during the FST (Figure 5A) and TST (Figure 5B), along with the prolonged latency to feed in the NSFT (Figure 5C), were dose-dependently reduced in mice treated with JNJ-38877605. In the OFT test, it was found that treatment with Met inhibitor JNJ-38877605 increased the distance traveled in the test field (Figure 5D), the distance traveled in the central area of the test field (Figure 5E), and the number of crossing squares (Figure 5F) in STZ-induced diabetic mice in a dose-dependent manner. Moreover, JNJ-38877605 treatment at a dose of 20 mg/kg had no significant effect on the behavioral parameters tested in FST, TST, NSFT, and OFT in the control group (Figure 5A-F). These results indicated that the Met inhibitor alleviated STZ-induced depression-like behaviors.

As expected, treatment with the Met inhibitor JNJ-38877605 at a dose of 20 mg/kg significantly blocked the elevated phosphorylation of Met in the hippocampus of STZ-induced diabetic mice (Supplemental Figure S6B-C). JNJ-38877605 treatment also led to significant decreases in Src expression (Figure 5G) and the phosphorylation of Btk (Supplemental Figure S6D-E) in the hippocampus of STZ-induced diabetic mice. To investigate whether the Met inhibitor inactivated NF-κB in the microglia, double-immunofluorescence staining for Iba1 and phosphorylated NF-κB p65 subunit was performed. As shown in Figure 5H-I and Supplemental Figure S7, STZ-induced diabetic mice exhibited increased Iba1^+^ and phosphorylated p65 (p-p65)^+^ cells in the hippocampus, as compared to control mice. The quantification analysis showed that STZ treatment significantly increased the percentage of Iba1^+^/p-p65^+^ cells in total Iba1^+^ cells as compared to the control, which was largely reversed by the Met inhibitor JNJ-38877605.

We then investigated the effect of the Met inhibitor on the number of Iba1^+^ microglia in three subregions of the hippocampus. The results showed that treatment with the Met inhibitor JNJ-38877605 significantly decreased the numbers of Iba1^+^ cells in the CA1, CA2/3, and DG subregions of the hippocampus in the STZ-induced diabetic mice (Figure 5J, Supplemental Figure S8). In addition, qRT-PCR results demonstrated that the STZ-induced mRNA expression of Iba1 (Figure 5K), as well as pro-inflammatory mediators including TNF-α, IL-6, CCL2, and iNOS, were also markedly reduced in the hippocampus by treatment with the Met inhibitor JNJ-38877605 (Figure 5L). Taken together, these findings indicated that the Met inhibitor inactivates Met/Src/Btk/NF-κB signaling pathways and attenuates depression-like behaviors, microglia activation, and neuroinflammation in STZ-induced diabetic mice.

### Effects of Met inhibitor or KLK8 deficiency on hippocampal neuroplasticity in STZ-induced diabetic mice

**Figure 6:**
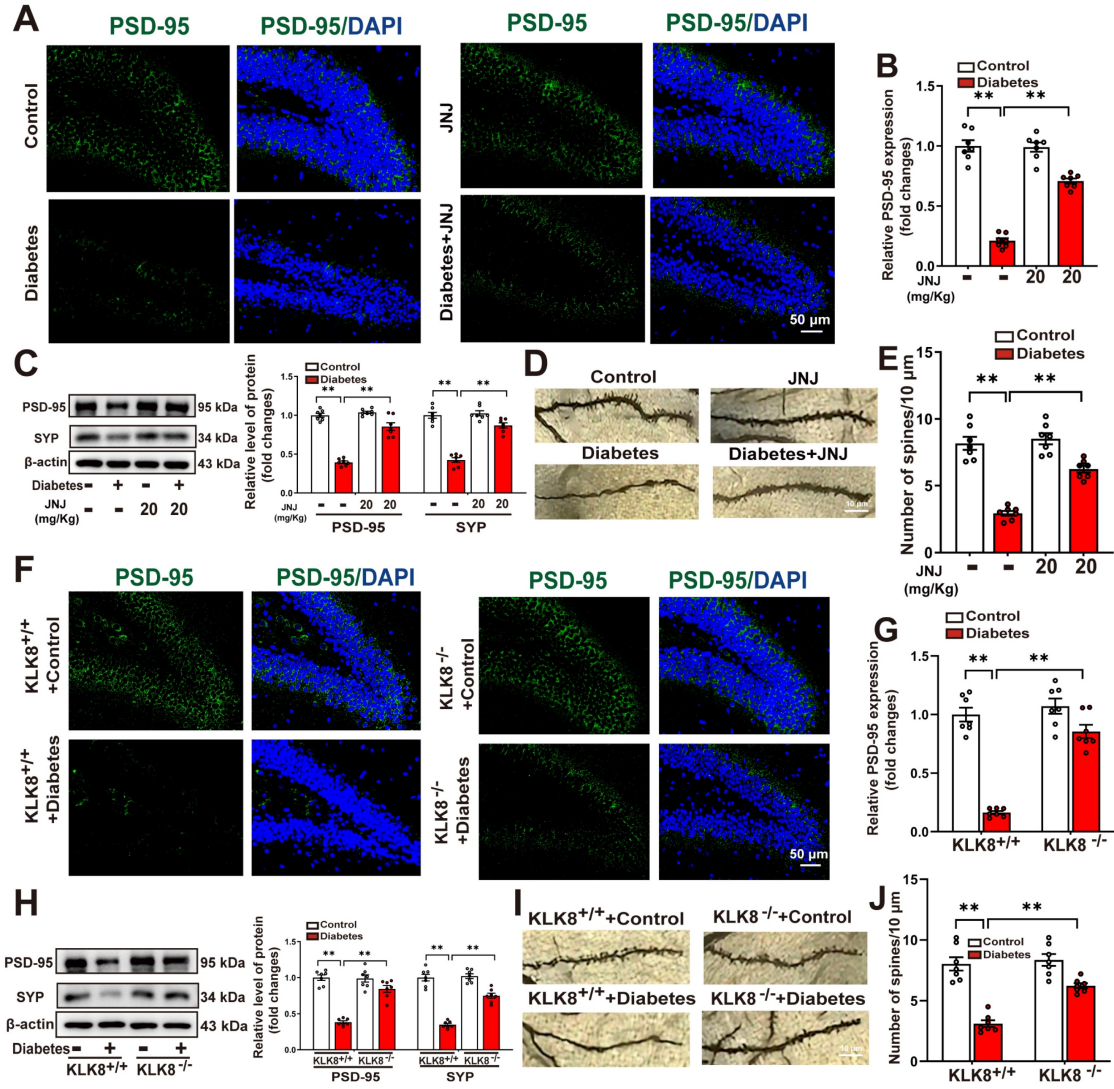
** Effects of Met inhibitor or KLK8 deficiency on hippocampal neuroplasticity in STZ-induced diabetic mice.** Control or STZ-induced diabetic mice were subjected to moderate intensity treadmill training for 5 weeks. **A** and** F**, Hippocampal sections were stained with fluorophore-labeled antibodies against PSD-95 (green). DAPI staining was used to detect nuclei (blue). Scale bar = 50 μm. **B** and** G**, The quantification of the fluorescence intensity of PSD-95 (B, F_1,24 (Met inhibitor)_ = 50.246, *p* < 0.001; F_1,24 (treatment)_ = 243.038, *p* < 0.001; F_1,24 (Met inhibitor×treatment)_ =54.295 , *p* < 0.001. G, F_1,24 (genotype)_ = 52.468, *p* < 0.001; F_1,24 (treatment)_ = 99.466, *p* < 0.001; F_1,24 (genotype×treatment)_ = 34.622, *p* < 0.001). **C** and** H**, protein expression levels of PSD-95 (C, F_1,24 (Met inhibitor)_ = 31.067, *p* < 0.001; F_1,24 (treatment)_ = 89.132, *p* < 0.001; F_1,24 (Met inhibitor×treatment)_ = 34.285, *p* < 0.001. H , F_1,24 (genotype)_ = 67.412, *p* < 0.001; F_1,24 (treatment)_ = 169.259, *p* < 0.001; F_1,24 (genotype×treatment)_ = 48.346, *p* < 0.001) and SYP (C, F_1,24 (Met inhibitor)_ = 37.682, *p* < 0.001; F_1,24 (treatment)_ = 180.341, *p* < 0.001; F_1,24 (Met inhibitor×treatment)_ = 30.558, *p* < 0.001. H, F_1,24 (genotype)_ = 49.441, *p* < 0.001; F_1,24 (treatment)_ = 119.223, *p* < 0.001; F_1,24 (genotype×treatment)_ = 39.381, *p* < 0.001) were detected by western blotting. Representative protein bands were presented on the left of the histograms. **D** and** I**, Representative microphotograph of Golgi-Cox staining in the hippocampal sections. Scale bar = 10 μm. **E** and** J**, Quantification of dendritic spine density of neurons in the hippocampus (E, F_1,24 (Met inhibitor)_ = 25.859, *p* < 0.001; F_1,24 (treatment)_ = 109.214, *p* < 0.001; F_1,24 (Met inhibitor×treatment)_ = 17.078, *p* < 0.001. J, F_1,24 (genotype)_ = 20.743, *p* < 0.001; F_1,24 (treatment)_ = 73.995, *p* < 0.001; F_1,24 (genotype×treatment)_ = 11.577, *p* = 0.002) . Data were presented as means ± SEM (n = 7, two-way ANOVA). ** *p* < 0.01. JNJ represents JNJ-38877605.

A growing body of evidence suggests that microglial activation plays a critical role in the development of abnormal neuroplasticity associated with depression 33-35. To further investigate the effects of Met inhibition or KLK8 deficiency on hippocampal neuroplasticity, we assessed synaptic plasticity and dendritic spine formation using immunofluorescence labeling, western blot analysis, and Golgi staining. Our immunofluorescence analysis demonstrated a significant reduction in the mean fluorescence intensity of postsynaptic density protein-95 (PSD-95) in the hippocampus of STZ-induced diabetic mice compared to control mice. This impairment was significantly ameliorated by either Met inhibitor treatment (Figure 6A-B) or KLK8 deficiency (Figure 6F-G). Western blot analysis confirmed that both interventions effectively reversed the diabetes-induced downregulation of synaptophysin (SYP) and PSD-95, which are key markers for evaluating synaptic integrity and plasticity (Figure 6C, Figure 6H).

We subsequently examined whether Met inhibitor treatment or KLK8 deficiency influenced dendritic spine formation in hippocampal neurons using Golgi staining. As illustrated in Figure 6D-E&Figure 6I-J, STZ-induced diabetic mice exhibited a significant reduction in the number of spines in hippocampal neurons compared to control mice. Both Met inhibitor treatment (Figure 6D-E) and KLK8 deficiency (Figure 6I-J) markedly restored spine density in these neurons. These findings suggest that the KLK8/Met signaling pathway plays a critical role in diabetes-induced synaptic plasticity deficits in the hippocampus.

### Running exercise reverses KLK8 upregulation and inactivates Met/Src/Btk/NF-κB signaling pathways, thereby attenuating microglia activation and neuroinflammation in the hippocampus of STZ-induced diabetic mice

**Figure 7:**
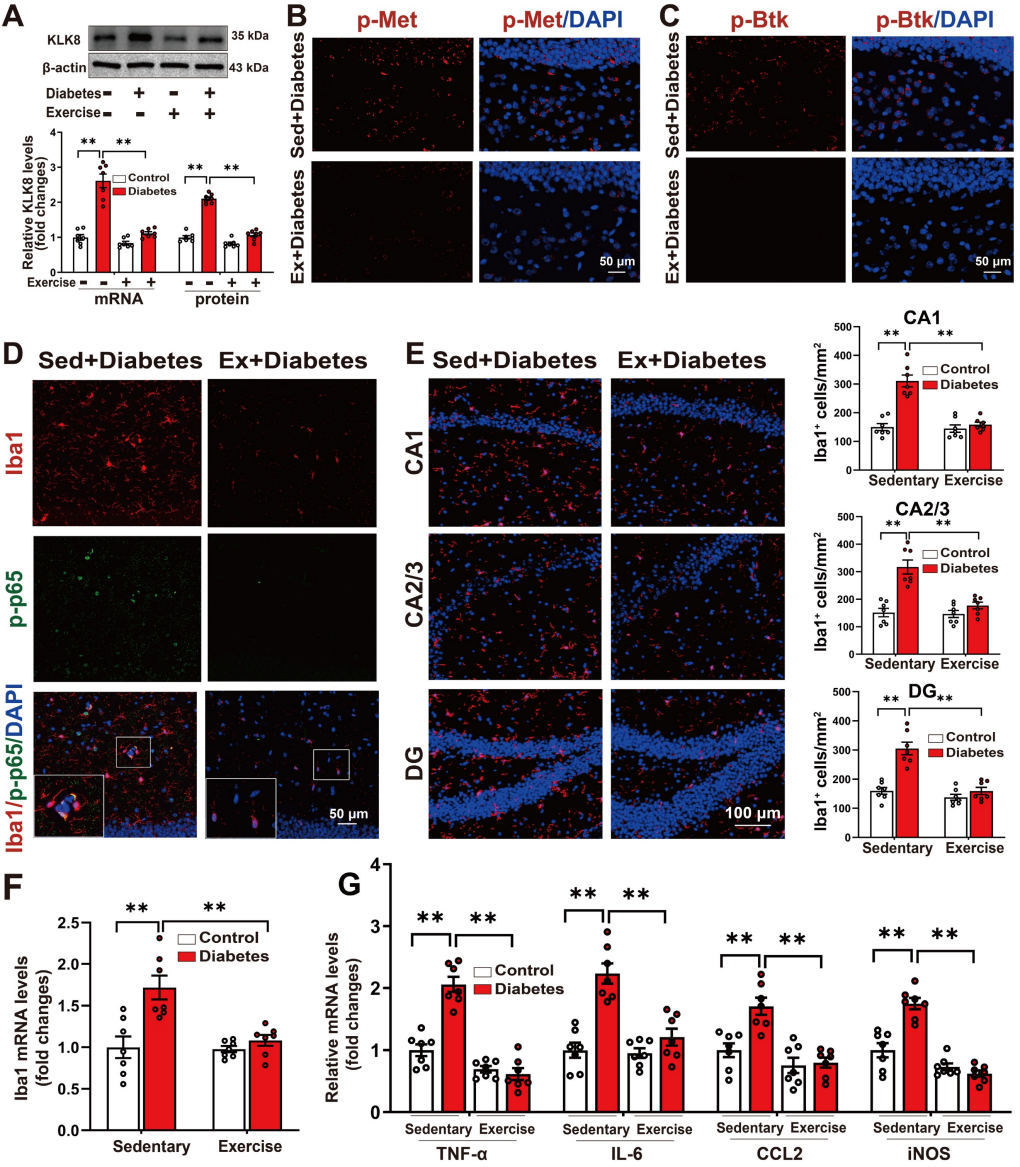
** Running exercise reverses KLK8 upregulation and inactivates Met/Src/Btk/NF-κB signaling pathways, thereby attenuating microglia activation and neuroinflammation in the hippocampus of STZ-induced diabetic mice.** Control or STZ-induced diabetic mice were subjected to moderate intensity treadmill training for 5 weeks. **A,** The levels of KLK8 mRNA and protein were measured in the hippocampus of the STZ-induced diabetic mice subjected to sedentary conditions or running exercise by qRT-PCR (F_1,24 (running exercise)_ = 57.427, *p* < 0.001; F_1,24 (treatment)_ = 74.348, *p* < 0.001; F_1,24 (running exercise×treatment)_ = 37.177, *p* < 0.001) and western blotting F_1,24 (running exercise)_ = 157.255, *p* < 0.001; F_1,24 (treatment)_ = 195.184, *p* < 0.001; F_1,24 (running exercise×treatment)_ = 78.996, *p* < 0.001), respectively. Representative protein bands were presented on the top of the histograms. **B,** Hippocampal sections were stained with fluorophore-labeled antibodies against phosphorylated Met (p-Met, red). DAPI staining was used to detect nuclei (blue). Scale bar = 50 μm. **C,** Hippocampal sections were stained with fluorophore-labeled antibodies against phosphorylated Btk (p-Btk, red). DAPI staining was used to detect nuclei (blue). Scale bar = 50 μm. **D,** Hippocampal sections were stained with fluorophore-labeled antibodies against the microglial cell marker Iba1 (red) and p-p65 (green). DAPI staining was used to detect nuclei (blue). The merge image represents double positive staining for Iba1 and p-p65. Areas in white boxes were shown enlarged. Scale bar = 50 μm. **E,** Immunofluorescent staining showed Iba1 (red) expression in the CA1, CA2/3, and DG subregions of the hippocampus. Nuclei were counterstained with DAPI (blue). Scale bar = 100 μm. The quantification of Iba1^+^ cell numbers in each subfield of the hippocampus were presented in the right panels (CA1: F_1,24 (running exercise)_ = 30.348, *p* < 0.001; F_1,24 (treatment)_ = 37.189, *p* < 0.001; F_1,24 (running exercise×treatment)_ = 26.515, *p* < 0.001. CA2/3: F_1,24 (running exercise)_ = 17.621, *p* < 0.001; F_1,24 (treatment)_ = 32.211, *p* < 0.001; F_1,24 (running exercise×treatment)_ = 15.279, *p* < 0.001. DG: F_1,24 (running exercise)_ = 33.171, *p* < 0.001; F_1,24 (treatment)_ = 32.722, *p* < 0.001; F_1,24 (running exercise×treatment)_ = 17.916, *p* < 0.001). **F,** The mRNA expression levels of Iba1 in the hippocampus were detected by qRT-PCR (F_1,24 (running exercise)_ = 9.947, *p* = 0.004; F_1,24 (treatment)_ = 15.644, *p* < 0.001; F_1,24 (running exercise×treatment)_ = 8.656, *p* < 0.007). **G,** The mRNA expression levels of TNF-α, IL-6, CCL2, and iNOS in the hippocampus were detected by qRT-PCR (TNF-α: F_1,24 (running exercise)_ = 85.403, *p* < 0.001; F_1,24 (treatment)_ = 28.816, *p* < 0.001; F_1,24 (running exercise×treatment)_ = 36.099, *p* < 0.001. IL-6: F_1,24 (running exercise)_ =17.182, *p* < 0.001; F_1,24 (treatment)_ = 33.374,* p* < 0.001; F_1,24 (running exercise×treatment)_ = 14.296, *p* < 0.001. CCL2: F_1,24 (running exercise)_ = 24.974, *p* < 0.001; F_1,24 (treatment)_ = 10.641,* p* = 0.003; F_1,24 (running exercise×treatment)_ = 8.261, *p* = 0.008. iNOS: F_1,24 (running exercise)_ = 71.497, *p* < 0.001; F_1,24 (treatment)_ = 15.102, *p* < 0.001; F_1,24 (running exercise×treatment)_ = 27.053, *p* < 0.001). Data were presented as means ± SEM (n = 7, two-way ANOVA). ** *p* < 0.01.

Several studies have demonstrated that exercise training can ameliorate behavioral abnormalities associated with various mental disorders, including anxiety and depression 14, 36-42. To explore the effects of aerobic exercise on hippocampal KLK8 expression and the depression-like behavior in the STZ-induced diabetic mice, we subjected both control and STZ-induced diabetic mice to five weeks of aerobic treadmill training (Supplemental Figure S9A). We found that aerobic exercise training significantly reduced the mRNA and protein expression levels of KLK8 in the hippocampal tissues of STZ-induced diabetic mice (Figure 7A).

It was discovered that running exercise training gave rise to significant declines in the phosphorylation of Met (Figure 7B, Supplemental Figure S9B-C) and Btk (Figure 7C, Supplemental Figure S9D-E), as well as Src expression in the hippocampus of STZ-induced diabetic mice (Supplemental Figure S9F). The quantification analysis of double-immunofluorescence staining for Iba1 and phosphorylated NF-κB p65 subunit demonstrated that exercise training notably decreased the percentage of Iba1^+^/p-p65^+^ cells in total Iba1^+^ cells in the hippocampus of STZ-induced diabetic mice (Figure 7D, Supplemental Figure S10). These findings indicated that running exercise inactivates Met/Src/Btk/NF-κB signaling pathways in the hippocampus of STZ-induced diabetic mice.

We then examined the effect of exercise training on the number of Iba1^+^ microglia in three subregions of the hippocampus. The results indicated that aerobic exercise training significantly decreased the numbers of Iba1^+^ cells in the CA1, CA2/3, and DG subregions of the hippocampus in the STZ-induced diabetic mice (Figure 7E, Supplemental Figure S11). Additionally, qRT-PCR results manifested that the STZ-induced mRNA expression levels of Iba1 (Figure 7F), as well as pro-inflammatory mediators including TNF-α, IL-6, CCL2, and iNOS in the hippocampus were also markedly mitigated by aerobic exercise training (Figure 7G). Taken together, these findings suggest that aerobic exercise reverses KLK8 upregulation and mitigates depression-like behaviors, microglia activation, and neuroinflammation in STZ-induced diabetic mice.

### Running exercise improves hippocampal neuroplasticity and alleviates depression-like behaviors in STZ-induced diabetic mice

**Figure 8:**
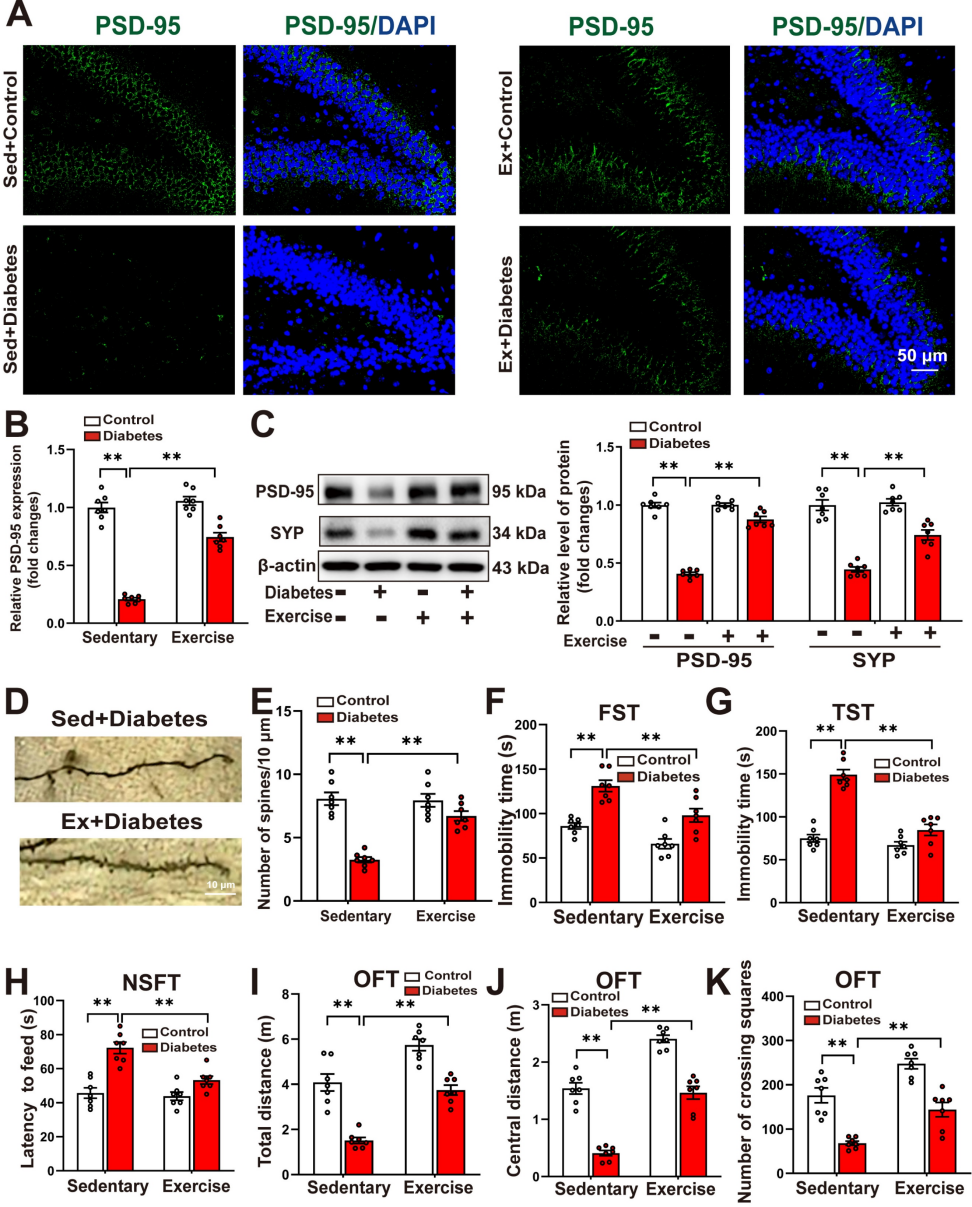
** Running exercise improves hippocampal neuroplasticity and alleviates depression-like behaviors in STZ-induced diabetic mice.** Control or STZ-induced diabetic mice were subjected to moderate intensity treadmill training for 5 weeks. **A,** Hippocampal sections were stained with fluorophore-labeled antibodies against PSD-95 (green). DAPI staining was used to detect nuclei (blue). Scale bar = 50 μm. **B,** The quantification of the fluorescence intensity of PSD-95 (F_1,24 (running exercise)_ = 73.555, *p* < 0.001; F_1,24 (treatment)_ = 253.885, *p* < 0.001; F_1,24 (running exercise×treatment)_ = 47.885, *p* < 0.001). **C,** protein expression levels of PSD-95 (F_1,24 (running exercise)_ = 140.206, *p* < 0.001; F_1,24 (treatment)_ = 327.622, *p* < 0.001; F_1,24 (running exercise×treatment)_ = 136.337, *p* < 0.001) and SYP (F_1,24 (running exercise)_ = 19.705, *p* < 0.001; F_1,24 (treatment)_ = 133.349, *p* < 0.001; F_1,24 (running exercise×treatment)_ = 14.086, *p* < 0.001) were detected by western blotting. Representative protein bands were presented on the left of the histograms. **D,** Representative microphotograph of Golgi-Cox staining in the hippocampal sections. Scale bar = 10 μm. **E,** Quantification of dendritic spine density of neurons in the hippocampus (F_1,24 (running exercise)_ = 15.754, *p* < 0.001; F_1,24 (treatment)_ = 51.924, *p* < 0.001; F_1,24 (running exercise×treatment)_ = 18.282, *p* < 0.001). **F-K,** Depressive behavioral tests were performed in the STZ-induced diabetic mice subjected to sedentary conditions or running exercise. **F,** The immobility time in the FST (F_1,24 (running exercise)_ = 20.277, *p* < 0.001; F_1,24 (treatment)_ = 42.65, *p* < 0.001; F_1,24 (running exercise×treatment)_ = 1.196, *p* = 0.285). **G,** The immobility time in the TST (F_1,24 (running exercise)_ = 47.15, *p* < 0.001; F_1,24 (treatment)_ = 76.039, *p* < 0.001; F_1,24 (running exercise×treatment)_ = 28.555, *p* < 0.001). **H,** The latency to feed in the NSFT (F_1,24 (running exercise)_ = 13.423, *p* = 0.001; F_1,24 (treatment)_ = 39.99,* p* < 0.001; F_1,24 (running exercise×treatment)_ = 9.068, *p* = 0.006). **I,** Total distance in the OFT (F_1,24 (running exercise)_ = 55.501, *p* < 0.001; F_1,24 (treatment)_ = 77.358,* p* < 0.001; F_1,24 (running exercise×treatment)_ = 1.188, *p* = 0.287).** J,** Central distance in the OFT (F_1,24 (running exercise)_ = 129.104, *p* < 0.001; F_1,24 (treatment)_ = 150.511, *p* < 0.001; F_1,24 (running exercise×treatment)_ = 1.244, *p* < 0.276). **K,** Number of crossing squares in the OFT (F_1,24 (running exercise)_ = 31.274, *p* < 0.001; F_1,24 (treatment)_ = 64.746, *p* < 0.001; F_1,24 (running exercise×treatment)_ = 0.027, *p* < 0.872). Data were presented as means ± SEM (n = 7, two-way ANOVA). ** *p* < 0.01.

We further examined the effects of running exercise training on hippocampal neuroplasticity. Immunofluorescence analysis revealed that aerobic exercise training significantly reversed the STZ-induced reduction in the mean fluorescence intensity of PSD-95 in the hippocampus (Figure 8A-B). Western blot analysis confirmed that running exercise markedly restored the diabetes-induced downregulation of SYP and PSD-95 (Figure 8C). Moreover, Golgi staining demonstrated that running exercise effectively reversed the diabetes-induced decrease in dendritic spine density in hippocampal neurons of STZ-induced diabetic mice (Figure 8D-E, Supplemental Figure S12A-B).

As depicted in Figure 8F-H, the STZ-induced increases in the immobility time in the FST (Figure 8F) and the TST (Figure 8G), as well as the increases in the latency to feed in the NSFT (Figure 8H), were significantly decreased in running exercise-trained mice. In the OFT test, we discovered that running exercise training increased the distance traveled in the test field (Figure 8I), the distance traveled in the central area of the test field (Figure 8J), and the number of crossing squares (Figure 8K) in STZ-induced diabetic mice.

These findings suggest that running exercise enhances hippocampal neuroplasticity and mitigates depression-like behaviors in STZ-induced diabetic mice.

## Discussion

Several clinical studies have reported the positive correlation between systemic inflammation and depressive symptoms in patients with type 1 and type 2 diabetes 43-45. Notably, a recent study indicated that type 2 diabetic patients with insufficient physical activity or high systemic immune-inflammation index levels were significantly more prone to have higher stress, anxiety, and depression 46. Indeed, laboratory studies have achieved significant advancements in uncovering the contribution of microglial activation and neuroinflammation to the onset of diabetes-associated depression 3, 4. However, the mechanism underlying the diabetes-induced neuroinflammation remains largely unexplored. By integrating the public gene-chip data and our validation results, we identified that KLK8 was upregulated in the hippocampus of diabetic mice and high glucose-treated microglial cells. KLK8 (also known as neuropsin) is highly expressed in the hippocampus, amygdala, and other brain regions related to emotion management, and plays a crucial role in neuroplasticity, learning, and memory processes 7-11. Previous studies have demonstrated that KLK8 inactivation protected against chronic stress-induced depression-like behaviors by alleviating hippocampal glutamate dysregulation 10 and neuronal apoptosis 11. In the present study, we demonstrated for the first time that hyperglycemia-induced KLK8 upregulation per se was sufficient to induce microglial activation and neuroinflammation. In addition, the inactivation of KLK8 by exercise training or genetic knockout of KLK8 significantly mitigated hippocampal microglial activation, neuroinflammation, and depression-like behaviors in STZ-induced diabetic mice. These findings collectively emphasize the critical role of KLK8 in diabetic-induced neuroinflammation, while suggesting that the antidepressant effects provided by aerobic exercise training during diabetes in mice may be attributed at least partly to the inactivation of KLK8 and the consequent suppression of microglial activation and neuroinflammation.

The transcription profiling of KLK8-overexpressed microglial cells disclosed a crucial role for the Met signaling pathway in mediating the stimulatory effects of KLK8 on these cells. Met is a receptor tyrosine kinase (RTK) encoded by the Met gene and has HGF as its native peptide ligand, which is known to be secreted by microglial cells 27, 47. HGF is synthesized and secreted as an inactive precursor termed pro-HGF, with its active form generated through proteolytic cleavage of pro-HGF 29. *In vitro* studies have manifested that several secreted serine proteases, including urokinase, tissue-type plasminogen activator (tPA), HGF activator and various members of the KLK family such as KLK4, KLK5, and KLK14, can convert pro-HGF into mature HGF 30, 31, 48-50. The present study provides compelling evidence indicating that KLK8 plays a critical role in the proteolytic processing of pro-HGF, thereby mediating the high glucose-induced release of HGF from microglial cells. Furthermore, the application of an HGF-neutralizing antibody effectively inactivated the Met signaling pathway and inhibited microglial activation in both KLK8- or high glucose-treated microglial cells. These findings imply that the upregulation of KLK8 contributes to the enhanced stimulation of the HGF/Met signaling pathway and subsequent microglial activation under hyperglycemic conditions by directly mediating the proteolytic cleavage of pro-HGF.

Met is expressed in immune and glial cells and is implicated in cell migration, proliferation, chemotaxis, cytokine secretion, and antigen presentation 51-53. Many peripheral diseases, including skeletal muscle injury 54, rheumatoid arthritis 55, autoimmune heart disease 56, and ulcerative colitis 57, exhibit activation of the Met signaling pathway, but there has been contradictory evidence regarding whether it promotes or inhibits inflammation. Several groups have reported that Met stimulates the secretion of IL-10 and TGF-β1 and leads to tissue repair by shifting macrophage polarization towards the anti-inflammatory M2 phenotype 58, 59. In contrast, Met activation augments monocyte migration and chemokine secretion in rheumatoid arthritis 55. Elevations in Met^+^ T cells or neutrophils are found to exacerbate local inflammation and tissue damage during cardiac autoimmunity 56 and colitis 57, respectively. To date, limited studies have explored the contribution of the HGF/Met signaling pathway in the pathogenesis of non-cancer diseases in the central nervous system, but have yielded relatively consistent results. Met drives the proliferation of M1-polarized macrophages 60, and is regarded as an immune marker of highly pathogenic pro-inflammatory and pro-migratory CD4^+^ T lymphocytes associated with neuroinflammation during experimental autoimmune encephalomyelitis 52. Rehman et al report that HGF/Met triggers detrimental reactive microglia and neuroinflammation through activating the Btk/NFκB signaling pathway in traumatic brain injury 27. Our *in vitro* studies manifested that KLK8/high glucose-induced microglial activation could be blocked by either HGF-neutralizing antibody or Met inhibitor. Additionally, systemic administration of Met inhibitor effectively attenuated hippocampal microglia activation and neuroinflammation and prevented the depression-like behaviors in diabetic mice. These findings imply that the contribution of the HGF/Met signaling to the regulation of inflammatory responses varies in significance depending on the local microenvironment and cell type. Inactivation of the HGF/Met signaling pathway might be an effective strategy to inhibit microglia activation and neuroinflammation during diabetes.

Microglia are specialized brain-resident macrophages that integrate information about the brain and immune context under inflammatory conditions 61, 62. Notably, a recent study demonstrates that overexpression of KLK8 significantly increased Iba-1+/iNOS+ microglia, thereby exacerbating neuroinflammation and behavioral deficits in a rat model of intracranial hemorrhage 63. Inhibition of Btk signaling in myeloid cells induces a phenotypic switch in adipose tissue macrophages from a pro-inflammatory state to a pro-resolution phenotype, effectively reducing both local and systemic inflammation in diabetic mice 64. NF-κB activation has been widely recognized as a critical factor in the induction of microglial activation across various brain pathologies, including diabetic encephalopathy 65, 66. Recent studies indicate that running exercise decreases Iba-1+/iNOS+ microglia or Iba-1+/CD68+ microglia and alleviates neuroinflammation in the hippocampus of murine models of HFD-induced type 2 diabetes and chronic unpredictable stress-induced depression 41, 67. Taken together with the evidence provided by the present study that exercise training suppresses microglial KLK8/Met/Btk/NF-κB activation in the hippocampus of STZ-induced diabetic mice, these findings suggest that inactivation of KLK8 may contribute to the antidepressant effects of exercise by preserving the microglial homeostatic state in the hippocampus.

It is widely acknowledged that microglia provide protection and nutritional support to neurons, maintain neuronal homeostasis, and regulate synaptogenesis under normal physiological conditions. In response to various pathological stimuli, activated microglia progressively transition from supporting and repairing neurons to contributing to neuronal dysfunction 68. A growing body of evidence indicates that microglial activation plays a critical role in abnormal neuroplasticity during the development of depression 33-35. Consistent with these findings, we demonstrated that KLK8 deficiency, Met inhibition, and exercise training not only suppressed microglial activation and neuroinflammation but also enhanced neuroplasticity in the hippocampus of diabetic mice. Previous studies have shown that KLK8 inhibition or KLK8 deficiency counteracts defects in neuroplasticity under pathological conditions such as Alzheimer's disease and chronic stress 10, 69, 70. *In vitro*, treatment with KLK8 impairs proliferation and differentiation of neuronal cells and reduces the expression of neuroplasticity-supporting proteins such as EPHB2 70. Conversely, inhibition of excess KLK8 enhances proliferation, neurite density, neuronal soma size, and increases EPHB2 expression in neurons 70. Collectively, these findings suggest that KLK8 upregulation may contribute to hippocampal neuroplasticity deficits in diabetic mice either through direct effects on neurons or indirectly by promoting microglial activation.

The primary limitation of this study lies in the lack of clarification regarding the mechanisms governing KLK8 expression regulation under hyperglycemic conditions. Indeed, clinical studies have manifested significantly elevated circulating levels of KLK8 in diabetic individuals 71. With respect to individuals with depressive disorders, previous studies have indicated that peripheral blood KLK8 mRNA levels were markedly higher in patients with recurrent depressive disorders compared to either those with the first episode of depression or healthy controls 72, 73. These findings were further substantiated by an epigenome-wide association study of monozygotic twins, which identified that the depression symptomatology score was negatively associated with blood DNA methylation levels in the promoter region of KLK8 74. Notably, the cumulative evidence has emphasized the role of DNA methylation in the pathogenesis of behavioral impairment, including its function as an epigenetic transducer of the adaptive response to physical exercise 75-77. In the future, it will be crucial to collaborate with clinical experts to monitor circulating KLK8 levels in patients with diabetes-associated depression and investigate whether KLK8 levels correlate with DNA methylation levels in the KLK8 promoter region and the severity of depression symptomatology in diabetic patients.

## Conclusion

We documented for the first time that hyperglycemia-induced upregulation of KLK8 was a critical event capable of initiating microglial activation and neuroinflammation. Mechanistic exploration revealed that KLK8 upregulation contributed to the activation of the HGF/Met signaling pathway and subsequent microglial activation under hyperglycemic conditions by directly mediating the proteolytic cleavage of pro-HGF. Both Met inhibitor and KLK8 deficiency enhanced hippocampal neuroplasticity in STZ-induced diabetic mice. Additionally, running exercise reversed KLK8 upregulation and inactivated Met/Src/Btk/NF-κB signaling pathways, thereby attenuating neuroinflammation, improving neuroplasticity, and alleviating depression-like behaviors in diabetic mice. Thus, inhibiting the KLK8/Met pathway through genetic or pharmaceutical approaches to mitigate neuroinflammation may offer potential therapeutic strategies for diabetes-associated depression.

## Supplementary Material

Supplementary figures and tables.

Supplementary table.

## References

[1] ElSayed NA, Aleppo G, Aroda VR, Bannuru RR, Brown FM, Bruemmer D (2023). 2. Classification and Diagnosis of Diabetes: Standards of Care in Diabetes-2023. Diabetes care.

[2] Cooper ZW, O'Shields J, Ali MK, Chwastiak L, Johnson LCM (2024). Effects of Integrated Care Approaches to Address Co-occurring Depression and Diabetes: A Systematic Review and Meta-analysis. Diabetes Care.

[3] Su W-J, Li J-M, Zhang T, Cao Z-Y, Hu T, Zhong S-Y (2023). Microglial NLRP3 inflammasome activation mediates diabetes-induced depression-like behavior via triggering neuroinflammation. Prog Neuropsychopharmacol Biol Psychiatry.

[4] Yang F, Wang X, Qi J, Zhang K, Jiang Y, Feng B (2023). Glucagon-like Peptide 1 Receptor Activation Inhibits Microglial Pyroptosis via Promoting Mitophagy to Alleviate Depression-like Behaviors in Diabetic Mice. Nutrients.

[5] Li Q, Xie Y, Lin J, Li M, Gu Z, Xin T (2025). Microglia Sing the Prelude of Neuroinflammation-Associated Depression. Mol Neurobiol.

[6] Kaur M, Misra S, Swarnkar P, Patel P, Das Kurmi B, Das Gupta G (2023). Understanding the role of hyperglycemia and the molecular mechanism associated with diabetic neuropathy and possible therapeutic strategies. Biochem Pharmacol.

[7] Mella C, Figueroa CD, Otth C, Ehrenfeld P (2020). Involvement of Kallikrein-Related Peptidases in Nervous System Disorders. Front Cell Neurosci.

[8] Bukowski L, Chernomorchenko AMF, Starnawska A, Mors O, Staunstrup NH, Børglum AD (2020). Neuropsin in mental health. J Physiol Sci.

[9] Attwood BK, Bourgognon JM, Patel S, Mucha M, Schiavon E, Skrzypiec AE (2011). Neuropsin cleaves EphB2 in the amygdala to control anxiety. Nature.

[10] Chang S, Bok P, Sun CP, Edwards A, Huang GJ (2016). Neuropsin Inactivation Has Protective Effects against Depressive-Like Behaviours and Memory Impairment Induced by Chronic Stress. PLoS Genet.

[11] Xu D-H, Du J-K, Liu S-Y, Zhang H, Yang L, Zhu X-Y (2023). Upregulation of KLK8 contributes to CUMS-induced hippocampal neuronal apoptosis by cleaving NCAM1. Cell Death Dis.

[12] Luo J, Tang C, Chen X, Ren Z, Qu H, Chen R (2020). Impacts of Aerobic Exercise on Depression-Like Behaviors in Chronic Unpredictable Mild Stress Mice and Related Factors in the AMPK/PGC-1α Pathway. Int J Environ Res Public Healt.

[13] Li C, Xu X, Wang Z, Wang Y, Luo L, Cheng J (2020). Exercise ameliorates post-stroke depression by inhibiting PTEN elevation-mediated upregulation of TLR4/NF-κB/NLRP3 signaling in mice. Brain Res.

[14] Wang J, Carru C, Sedda S, Fiori PL, Li Z, Chen Z (2024). Comparative impact of exercise-based interventions for postpartum depression: A Bayesian network meta-analysis. Int J Gynaecol Obstet.

[15] Du JK, Yu Q, Liu YJ, Du SF, Huang LY, Xu DH (2021). A novel role of kallikrein-related peptidase 8 in the pathogenesis of diabetic cardiac fibrosis. Theranostics.

[16] Li DX, Wang CN, Wang Y, Ye CL, Jiang L, Zhu XY (2020). NLRP3 inflammasome-dependent pyroptosis and apoptosis in hippocampus neurons mediates depressive-like behavior in diabetic mice. Behav Brain Res.

[17] Yang L, Li D-X, Cao B-Q, Liu S-J, Xu D-H, Zhu X-Y (2021). Exercise training ameliorates early diabetic kidney injury by regulating the H2S/SIRT1/p53 pathway. FASEB J.

[18] Chen W, Wu S, Huang Y, Zhang T, Dong H, Zheng X (2021). A c-Met Inhibitor Suppresses Osteosarcoma Progression via the ERK1/2 Pathway in Human Osteosarcoma Cells. Onco Targets Ther.

[19] Hua Q, Li T, Liu Y, Shen X, Zhu X, Xu P (2021). Upregulation of KLK8 Predicts Poor Prognosis in Pancreatic Cancer. Front Oncol.

[20] Fu Q, Zhang YB, Shi CX, Jiang M, Lu K, Fu ZH (2024). GSDMD/Drp1 signaling pathway mediates hippocampal synaptic damage and neural oscillation abnormalities in a mouse model of sepsis-associated encephalopathy. J Neuroinflammation.

[21] Matsumoto-Miyai K, Ninomiya A, Yamasaki H, Tamura H, Nakamura Y, Shiosaka S (2003). NMDA-dependent proteolysis of presynaptic adhesion molecule L1 in the hippocampus by neuropsin. J Neurosci.

[22] Tamura H, Kawata M, Hamaguchi S, Ishikawa Y, Shiosaka S (2012). Processing of neuregulin-1 by neuropsin regulates GABAergic neuron to control neural plasticity of the mouse hippocampus. J Neurosci.

[23] Qian Y, Yang L, Chen J, Zhou C, Zong N, Geng Y (2024). SRGN amplifies microglia-mediated neuroinflammation and exacerbates ischemic brain injury. J Neuroinflammation.

[24] Luo W, Li Y, Zhao J, Niu R, Xiang C, Zhang M (2024). CD44-targeting hyaluronic acid-selenium nanoparticles boost functional recovery following spinal cord injury. J Nanobiotechnology.

[25] Raj S, Kesari KK, Kumar A, Rathi B, Sharma A, Gupta PK (2022). Molecular mechanism(s) of regulation(s) of c-MET/HGF signaling in head and neck cancer. Mol Cancer.

[26] Felix FB, Dias J, Vago JP, Martins DG, Beltrami VA, Fernandes DdO (2023). Blocking the HGF-MET pathway induces resolution of neutrophilic inflammation by promoting neutrophil apoptosis and efferocytosis. Pharmacol Res.

[27] Rehman R, Miller M, Krishnamurthy SS, Kjell J, Elsayed L, Hauck SM (2022). Met/HGFR triggers detrimental reactive microglia in TBI. Cell Rep.

[28] Fu J, Su X, Li Z, Deng L, Liu X, Feng X (2021). HGF/c-MET pathway in cancer: from molecular characterization to clinical evidence. Oncogene.

[29] Fukushima T, Uchiyama S, Tanaka H, Kataoka H (2018). Hepatocyte Growth Factor Activator: A Proteinase Linking Tissue Injury with Repair. Int J Mol Sci.

[30] Mukai S, Fukushima T, Naka D, Tanaka H, Osada Y, Kataoka H (2008). Activation of hepatocyte growth factor activator zymogen (pro-HGFA) by human kallikrein 1-related peptidases. FEBS J.

[31] Reid JC, Bennett NC, Stephens CR, Carroll ML, Magdolen V, Clements JA (2016). *In vitro* evidence that KLK14 regulates the components of the HGF/Met axis, pro-HGF and HGF-activator inhibitor 1A and 1B. Biol Chem.

[32] Kishi T, Cloutier SM, Kündig C, Deperthes D, Diamandis EP (2006). Activation and enzymatic characterization of recombinant human kallikrein 8. Biol Chem.

[33] Wang H, He Y, Sun Z, Ren S, Liu M, Wang G (2022). Microglia in depression: an overview of microglia in the pathogenesis and treatment of depression. J Neuroinflammation.

[34] Li Q, Xie Y, Lin J, Li M, Gu Z, Xin T (2025). Microglia Sing the Prelude of Neuroinflammation-Associated Depression. Mol Neurobiol.

[35] Fang S, Wu Z, Guo Y, Zhu W, Wan C, Yuan N (2023). Roles of microglia in adult hippocampal neurogenesis in depression and their therapeutics. Front Immunol.

[36] Wu J, Xu H, Wang S, Weng H, Luo Z, Ou G Regular exercise ameliorates high-fat diet-induced depressive-like behaviors by activating hippocampal neuronal autophagy and enhancing synaptic plasticity. Cell Death Dis. 20 24; 15: 737.

[37] Narita Z, Inagawa T, Stickley A, Sugawara N (2019). Physical activity for diabetes-related depression: A systematic review and meta-analysis. J Psychiatr Res.

[38] van der Feltz-Cornelis C, Allen SF, Holt RIG, Roberts R, Nouwen A, Sartorius N (2021). Treatment for comorbid depressive disorder or subthreshold depression in diabetes mellitus: Systematic review and meta-analysis. Brain Behav.

[39] Gilak-Dalasm M, Peeri M, Azarbayjani MA (2021). Swimming exercise decreases depression-like behaviour and inflammatory cytokines in a mouse model of type 2 diabetes. Exp Physiol.

[40] Zhou Z, Wang M, Huang C, Li Y, Gao L, Zhu Y (2022). Treadmill exercise training alleviates diabetes-induced depressive-like behavior and cognitive impairment by improving hippocampal CA1 neurons injury in db/db mice. Brain Res Bull.

[41] Xiao K, Luo Y, Liang X, Tang J, Wang J, Xiao Q (2021). Beneficial effects of running exercise on hippocampal microglia and neuroinflammation in chronic unpredictable stress-induced depression model rats. Transl Psychiatry.

[42] Liu L, Tang J, Liang X, Li Y, Zhu P, Zhou M (2024). Running exercise alleviates hippocampal neuroinflammation and shifts the balance of microglial M1/M2 polarization through adiponectin/AdipoR1 pathway activation in mice exposed to chronic unpredictable stress. Mol Psychiatry.

[43] Nguyen MM, Perlman G, Kim N, Wu C-Y, Daher V, Zhou A (2021). Depression in type 2 diabetes: A systematic review and meta-analysis of blood inflammatory markers. Psychoneuroendocrinology.

[44] Herder C, Hermanns N (2019). Subclinical inflammation and depressive symptoms in patients with type 1 and type 2 diabetes. Semin Immunopathol.

[45] Wang J, Zhou D, Dai Z, Li X (2021). Association Between Systemic Immune-Inflammation Index and Diabetic Depression. Clin Interv Aging.

[46] Rias YA, Tsai HT, Thato R, Apriyanto BS, Chou KR, Ho SC (2023). Synergistic Interactions of Insufficient Physical Activity and a High Systemic Immune-Inflammation Index on Psychological Problems in Indonesians With Type 2 Diabetes Mellitus. Biol Res Nurs.

[47] Yamagata T, Muroya K, Mukasa T, Igarashi H, Momoi M, Tsukahara T (1995). Hepatocyte Growth Factor Specifically Expressed in Microglia Activated RAS in the Neurons, Similar to the Action of Neurotrophic Factors. Biochem Biophys Res Commun.

[48] Oliveira AG, Araújo TG, Carvalho BdM, Rocha GZ, Santos A, Saad MJA (2018). The Role of Hepatocyte Growth Factor (HGF) in Insulin Resistance and Diabetes. Front Endocrinol (Lausanne).

[49] Tagirasa R, Yoo E (2022). Role of Serine Proteases at the Tumor-Stroma Interface. Front Immunol.

[50] Seillier C, Hélie P, Petit G, Vivien D, Clemente D, Le Mauff B (2022). Roles of the tissue-type plasminogen activator in immune response. Cell Immunol.

[51] Barbosa-Matos C, Borges-Pereira C, Libório-Ramos S, Fernandes R, Oliveira M, Mendes-Frias A (2024). Deregulated immune cell recruitment orchestrated by c-MET impairs pulmonary inflammation and fibrosis. Respir Res.

[52] Benkhoucha M, Tran NL, Breville G, Senoner I, Bradfield PF, Papayannopoulou T (2022). CD4+c-Met+Itgα4+ T cell subset promotes murine neuroinflammation. J Neuroinflammation.

[53] Takano T, Takano C, Funakoshi H, Bando Y (2024). Impact of Neuron-Derived HGF on c-Met and KAI-1 in CNS Glial Cells: Implications for Multiple Sclerosis Pathology. Int J Mol Sci.

[54] Lahmann I, Griger J, Chen J-S, Zhang Y, Schuelke M, Birchmeier C (2021). Met and Cxcr4 cooperate to protect skeletal muscle stem cells against inflammation-induced damage during regeneration. eLife.

[55] Hosonuma M, Sakai N, Furuya H, Kurotaki Y, Sato Y, Handa K (2021). Inhibition of hepatocyte growth factor/c-Met signalling abrogates joint destruction by suppressing monocyte migration in rheumatoid arthritis. Rheumatology (Oxford).

[56] Fanti S, Stephenson E, Rocha-Vieira E, Protonotarios A, Kanoni S, Shahaj E (2022). Circulating c-Met-Expressing Memory T Cells Define Cardiac Autoimmunity. Circulation.

[57] Stakenborg M, Verstockt B, Meroni E, Goverse G, De Simone V, Verstockt S (2020). Neutrophilic HGF-MET Signalling Exacerbates Intestinal Inflammation. J Crohns Colitis.

[58] Nishikoba N, Kumagai K, Kanmura S, Nakamura Y, Ono M, Eguchi H (2020). HGF-MET Signaling Shifts M1 Macrophages Toward an M2-Like Phenotype Through PI3K-Mediated Induction of Arginase-1 Expression. Front Immunol.

[59] Choi W, Lee J, Lee J, Lee SH, Kim S (2019). Hepatocyte Growth Factor Regulates Macrophage Transition to the M2 Phenotype and Promotes Murine Skeletal Muscle Regeneration. Front Physiol.

[60] Moransard M, Sawitzky M, Fontana A, Suter T (2010). Expression of the HGF receptor c-met by macrophages in experimental autoimmune encephalomyelitis. Glia.

[61] Zengeler KE, Lukens JR (2024). Microglia pack a toolbox for life. Trends Immunol.

[62] Paolicelli RC, Sierra A, Stevens B, Tremblay ME, Aguzzi A, Ajami B (2022). Microglia states and nomenclature: A field at its crossroads. Neuron.

[63] Tang L, Wang L, Jin F, Hao Y, Zhao T, Zheng W (2023). Inflammatory regulation by restraining M2 microglial polarization: Neurodestructive effects of Kallikrein-related peptidase 8 activation in intracerebral hemorrhage. Int Immunopharmacol.

[64] Purvis GSD, Collino M, van Dam AD, Einaudi G, Ng Y, Shanmuganathan M (2024). OxPhos in adipose tissue macrophages regulated by BTK enhances their M2-like phenotype and confers a systemic immunometabolic benefit in obesity. Diabetes.

[65] Zhang Y-y, Wang L, Guo H, Han T-t, Chang Y-h, Cui X-c (2023). Levetiracetam attenuates diabetes-associated cognitive impairment and microglia polarization by suppressing neuroinflammation. Front Pharmacol.

[66] Li R, Zhou Y, Zhang S, Li J, Zheng Y, Fan X (2022). The natural (poly)phenols as modulators of microglia polarization via TLR4/NF-κB pathway exert anti-inflammatory activity in ischemic stroke. Eur J Pharmacol.

[67] Xu H, Tian X, Wang Y, Lin J, Zhu B, Zhao C (2024). Exercise Promotes Hippocampal Neurogenesis in T2DM Mice via Irisin/TLR4/MyD88/NF-κB-Mediated Neuroinflammation Pathway. Biology (Basel).

[68] Pramanik S, Devi M H, Chakrabarty S, Paylar B, Pradhan A, Thaker M (2024). Microglia signaling in health and disease - Implications in sex-specific brain development and plasticity. Neurosci Biobehav Rev.

[69] Herring A, Münster Y, Akkaya T, Moghaddam S, Deinsberger K, Meyer J (2016). Kallikrein-8 inhibition attenuates Alzheimer's disease pathology in mice. Alzheimers Dement.

[70] Münster Y, Keyvani K, Herring A (2020). Inhibition of excessive kallikrein-8 improves neuroplasticity in Alzheimer's disease mouse model. Exp Neurol.

[71] Kobayashi H, Looker HC, Satake E, Saulnier PJ, Md Dom ZI, O'Neil K (2022). Results of untargeted analysis using the SOMAscan proteomics platform indicates novel associations of circulating proteins with risk of progression to kidney failure in diabetes. Kidney Int.

[72] Talarowska M, Bobińska K, Gaecka E, Szemraj J, Gaecki P (2016). Human neuropsin gene and social cognition in depression. Neuropsychiatry.

[73] Bobińska K, Mossakowska-Wójcik J, Szemraj J, Gałecki P, Zajączkowska M, Talarowska M (2017). Human neuropsin gene in depression. Psychiatr Danub.

[74] Starnawska A, Bukowski L, Chernomorchenko A, Elfving B, Müller HK, van den Oord E (2021). DNA methylation of the KLK8 gene in depression symptomatology. Clin Epigenetics.

[75] Tomiga Y, Sakai K, Ra S-G, Kusano M, Ito A, Uehara Y (2021). Short-term running exercise alters DNA methylation patterns in neuronal nitric oxide synthase and brain-derived neurotrophic factor genes in the mouse hippocampus and reduces anxiety-like behaviors. FASEB J.

[76] Qu Y, Zhai S, Zhang D, Li T, Xie Y, Tao S Moderating effects of clock genes DNA methylation on the relationship between physical activity trajectories and depressive symptoms among Chinese college students. Health Psychol, in press.

[77] Liao S, Tan M, Li M, Ren J, Wang Y, Zheng R (2022). Tai chi improves depressive symptoms among community-dwelling older persons by mediating BDNF methylation: A preliminary study. Geriatr Nurs.

