# Supplementary material for: KLK8/HGF/Met signaling pathway mediates diabetes-associated hippocampal neuroinflammation in male mice
Source: Theranostics. 2025 May 25;15(13):6290–312. doi: 10.7150/thno.109513 (PMC12159841; doi:10.7150/thno.109513)
Supplement: Supplementary file 1 — Supplementary figures and tables. [file thnov15p6290s1.pdf]

Supplemental Digital Content

KLK8/HGF/Met signaling pathway mediates diabetes-associated hippocampal neuroinflammation in male mice

Dan-Hong Xu[1#], Xiao-Yong Zhang[1#], Shi-Yu Liu[2#], Juan Wei[3], Jun-Hui Zhan[2], Jian-Kui Du[1], Yu-Jian Liu[2*], Xiao-Yan Zhu[1*]

1 Department of Physiology, Naval Medical University, Shanghai, 200433, China. 2 School of Kinesiology, The Key Laboratory of Exercise and Health Sciences of Ministry of Education, Shanghai University of Sport, Shanghai, 200438, China. 3 School of Sports and Health, Nanjing Sport Institute, Nanjing, 210014, China.

# Dan-Hong Xu, Xiao-Yong Zhang, Shi-Yu Liu contributed equally to this work.

* Corresponding authors: Prof. Xiao-Yan Zhu, Email: xiaoyanzhu@smmu.edu.cn; Prof. Yu-Jian Liu, Email: liuyujian@sus.edu.cn.

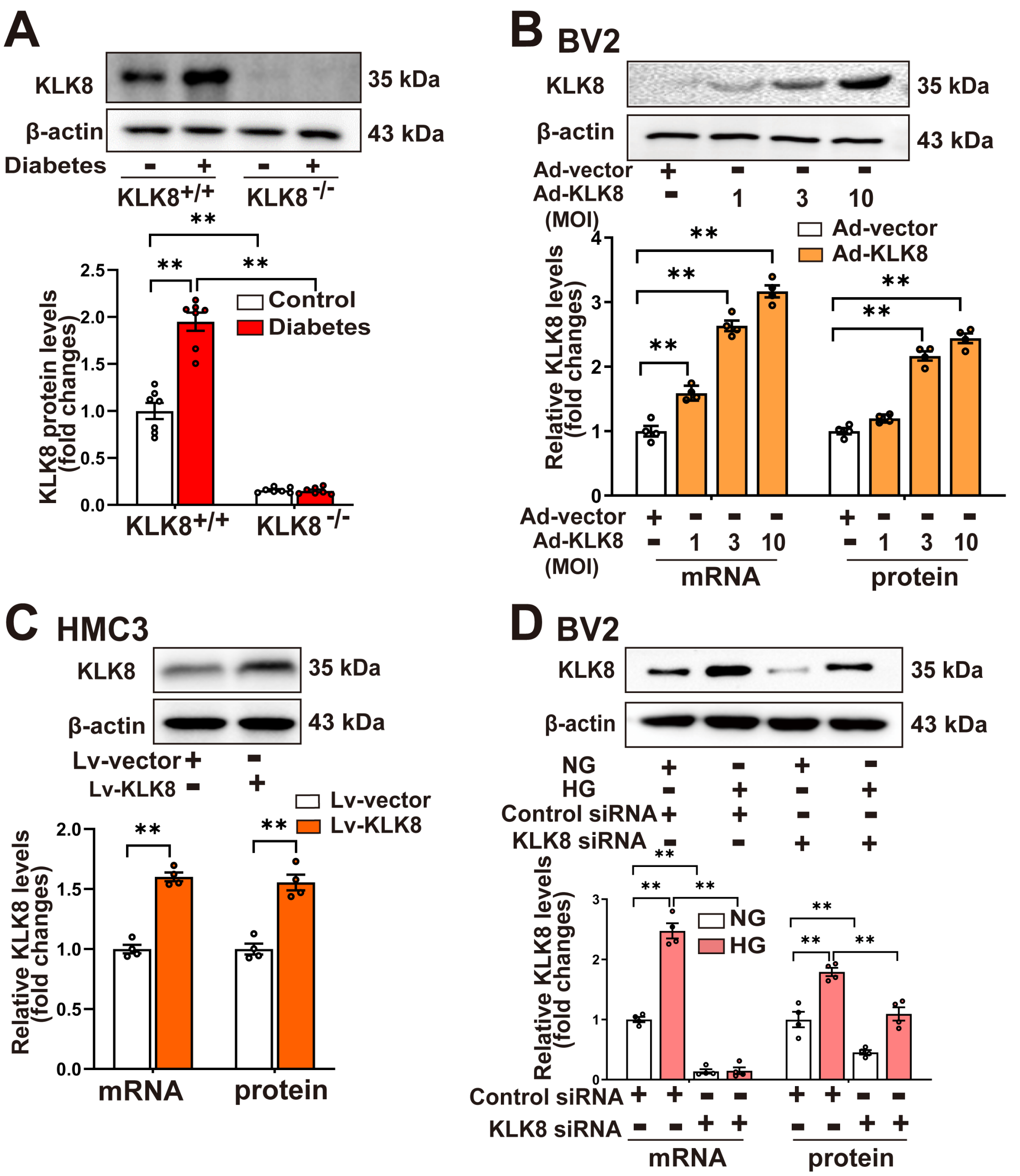

Supplemental Figure 1 Identification of the KLK8 expression in mouse hippocampal tissues, BV2 mouse microglial cells, and HMC3 human microglial cells (Related to Figure 1, Figure 2, and Figure 4). A, Hippocampal tissues were obtained from the KLK8-deficient ($KLK8^{-/-}$) and $KLK8^{+/+}$ mice after 5 weeks of STZ-induced diabetes. KLK8 protein levels were measured by western blotting (n = 7, two-way ANOVA, $F_{1,24\ (genotype)} = 407.999$, $p < 0.001$; $F_{1,24\ (treatment)} = 51.936$, $p < 0.001$; $F_{1,24\ (genotype \times treatment)} = 53.353$, $p < 0.001$). **B**, BV2 cells were infected with KLK8 adenovirus (Ad-KLK8) at a multiplicity of infection (MOI) of 1, 3, or 10 for 48 h. mRNA and protein expression levels of KLK8 were detected by qRT-PCR (n = 4, one-way ANOVA, $F_{3,12} = 154.762$, $p < 0.001$) and western blotting (n = 4, one-way ANOVA, $F_{3,12} = 145.039$, $p < 0.001$), respectively. **C**, A stably KLK8-overexpressing HMC3 cell line was generated through infection with KLK8 lentivirus (Lv-KLK8). Cells infected with an empty lentivirus served as the control group (Lv-Vector). The mRNA and protein levels of KLK8 were detected by qRT-PCR (n = 4, unpaired t-test, $p < 0.001$) and western blotting (n = 4, unpaired t-test, $p < 0.001$), respectively . **D**, BV2 cells were transfected with control siRNA or KLK8 siRNAs, and then treated with normal glucose (NG, 5.5 mM D-glucose) or high glucose (HG, 25 mM D-glucose) for 48 h. The mRNA and protein levels of KLK8 were examined by qRT-PCR (n = 4, one-way ANOVA, $F_{3,12} = 219.368$, $p < 0.001$) and western blotting (n = 4, one-way ANOVA, $F_{3,12} = 34.766$, $p < 0.001$), respectively. All

representative protein bands were presented on the top of the histograms. Data were presented as means ± SEM. ** $p < 0.01$.

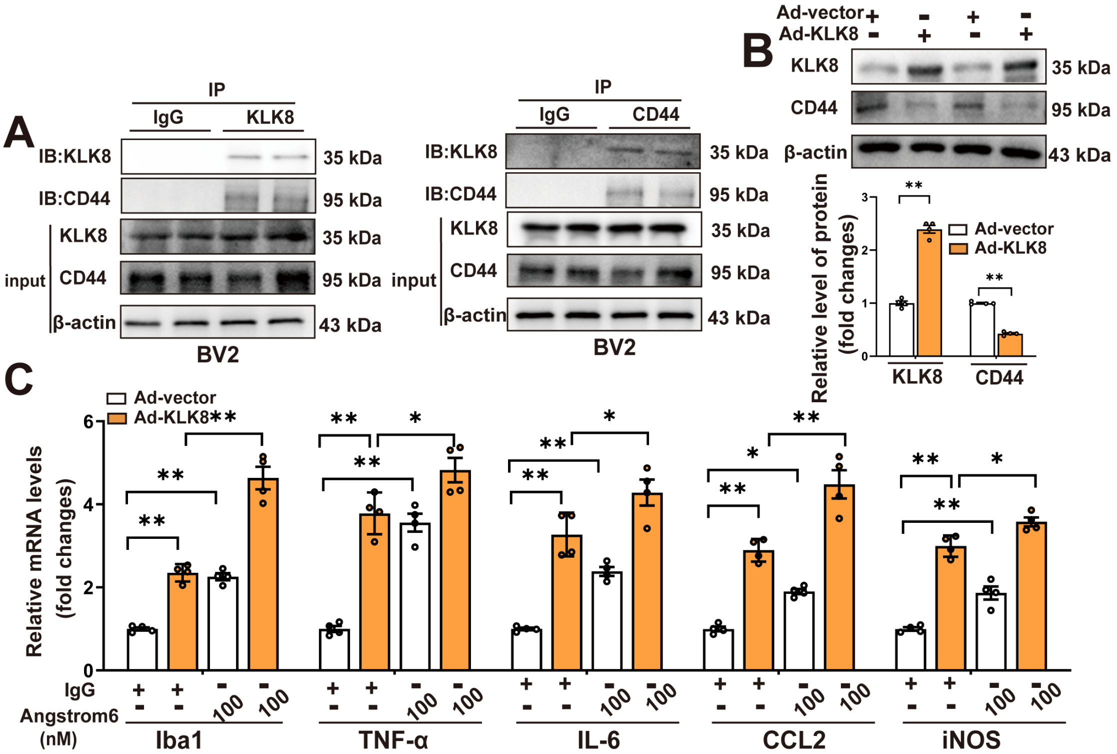

Supplemental Figure 2 Downregulation of CD44 is not the cause of microglial activation induced by Ad-KLK8. A, The association between CD44 and KLK8 was tested by reciprocal immunoprecipitations in BV2 cells. IgG was controlled for nonspecific interaction. **B,** BV2 cells were infected with Ad-Vector or Ad-KLK8 at a MOI of 3 for 48 h. Protein levels of KLK8 (n = 4, unpaired t-test, $p < 0.001$) and CD44 (n = 4, unpaired t-test, $p < 0.001$) in the BV2 cells were determined by western blot analysis. Representative protein bands were presented on the top of the histograms**. C,** BV2 cells were infected with Ad-KLK8 at a MOI of 3 for 48 h in the presence or absence of the CD44 activator Angstrom6 at the indicated concentrations. The mRNA expression levels of Iba1, TNF-α, IL-6, CCL2, and iNOS in the BV2 cells were detected by qRT-PCR (n = 4, one-way ANOVA, Iba1: $F_{3,12}$=97.617, $p < 0.001$. TNF-α: $F_{3,12}$= 52.4, $p < 0.001$. IL-6: $F_{3,12}$= 43.156, $p < 0.001$. CCL2: $F_{3,12}$= 62.658, $p < 0.001$. iNOS: $F_{3,12}$= 98.235, $p < 0.001$). Data were presented as means ± SEM. * p<0.05, ** $p < 0.01$.

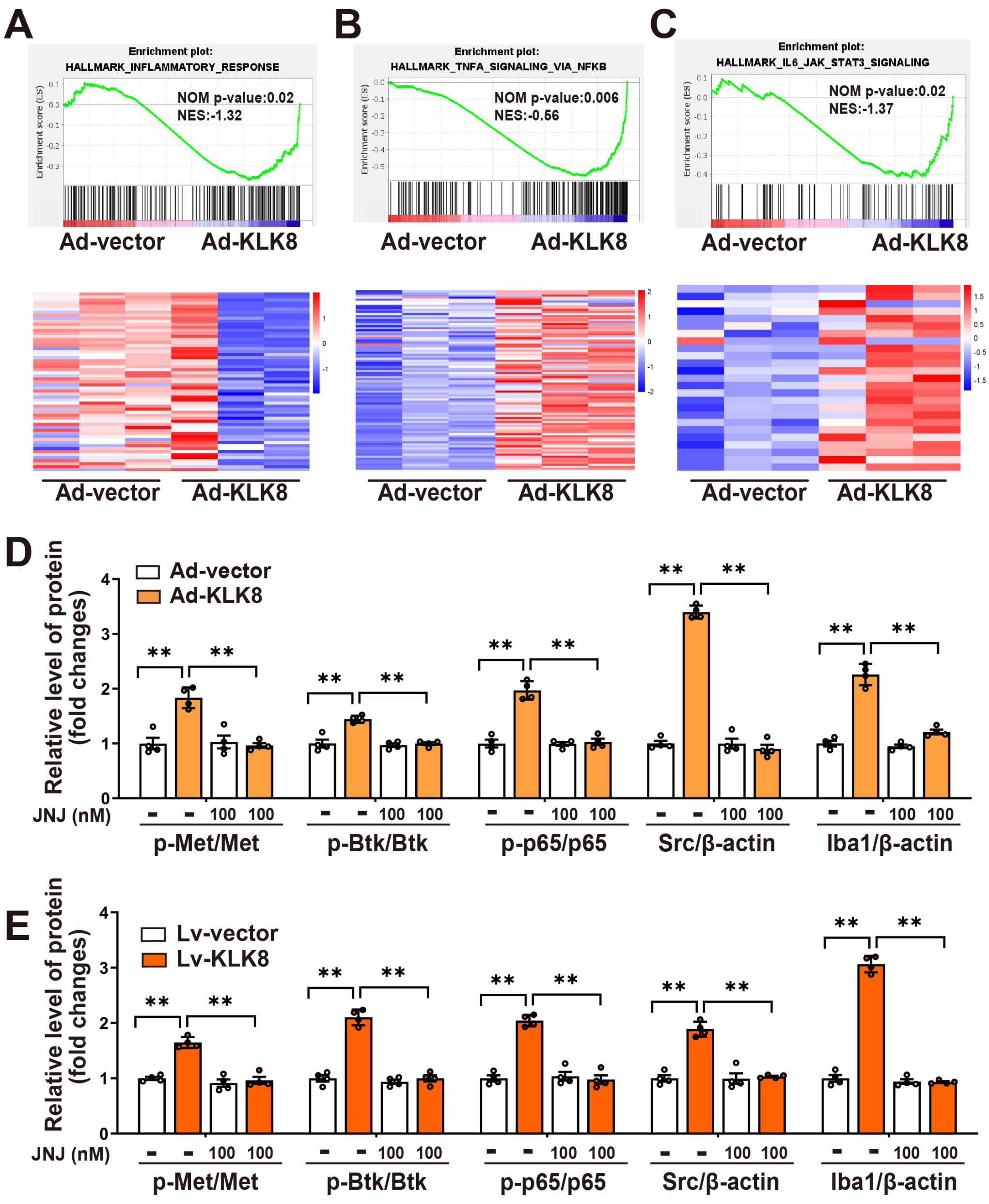

Supplemental Figure 3 KLK8 promotes microglial activation via a Met-dependent signaling pathway (Related to Figure 2). A-C, BV2 cells were infected with Ad-Vector or Ad-KLK8 at a MOI of 3 for 48 h. Dysregulated genes were analyzed by RNA-Seq. Gene set enrichment analysis (GSEA) was then performed to determine the enriched signaling pathways in KLK8-overexpressed BV2 cells. GSEA gene sets associated with Inflammatory response (**A**), TNFA signaling via NFκB (**B**) and IL6-JAK-STAT3 signaling (**C**) were significantly enriched in the Ad-KLK8-treated BV2 cells. Heat maps of the dysregulated target genes of the related signaling pathways were presented on the bottom of the GSEA plots. Red reflects upregulated and blue indicates downregulated genes. **D,** BV2 cells were infected with Ad-KLK8 at a MOI of 3 for 48 h in the presence or absence of the Met inhibitor JNJ-38877605 at the indicated concentrations. **E**, A stably KLK8-overexpressing HMC3 cell line was generated through infection with Lv-KLK8. Cells infected with an empty lentivirus served as the Lv-Vector. Stably KLK8-overexpressing HMC3 cells and control cells were treated with or without the Met inhibitor JNJ-38877605 at the indicated concentrations for 48 h. Protein levels of p-Met, Met, p-Btk, Btk, p-p65, p65, Src, and Iba1 in BV2 cells **(D)** or HMC3 cells **(E)** were determined by western blot analysis**.** Relative densitometry of the p-Met/Met, p-Btk/Btk, p-p65/p65, Src/β-actin and Iba1/β-actin protein band in BV2 cells **(**D**,** p-Met/Met: $F_{3,12}$ = 19.82, $p < 0.001$. p-Btk/Btk: $F_{3,12}$ = 25.616, $p < 0.001$. p-p65/p65: $F_{3,12}$ = 56.414, $p < 0.001$. Src/β-actin: $F_{3,12}$ = 298.381, $p < 0.001$. Iba1/β-actin: $F_{3,12}$ = 100.825, $p < 0.001$**)** and HMC3 cells **(**E**,** p-Met/Met: $F_{3,12}$

= 42.102, $p < 0.001$. p-Btk/Btk: $F_{3,12} = 98.25$, $p < 0.001$. p-p65/p65: $F_{3,12} = 60.308$, $p < 0.001$. Src/β-actin: $F_{3,12} = 44.647$, $p < 0.001$. Iba1/β-actin: $F_{3,12} = 390.789$, $p < 0.001$) were shown in bar graphs (n = 4, one-way ANOVA). Data were presented as means ± SEM. ** $p < 0.01$. JNJ represents JNJ-38877605.

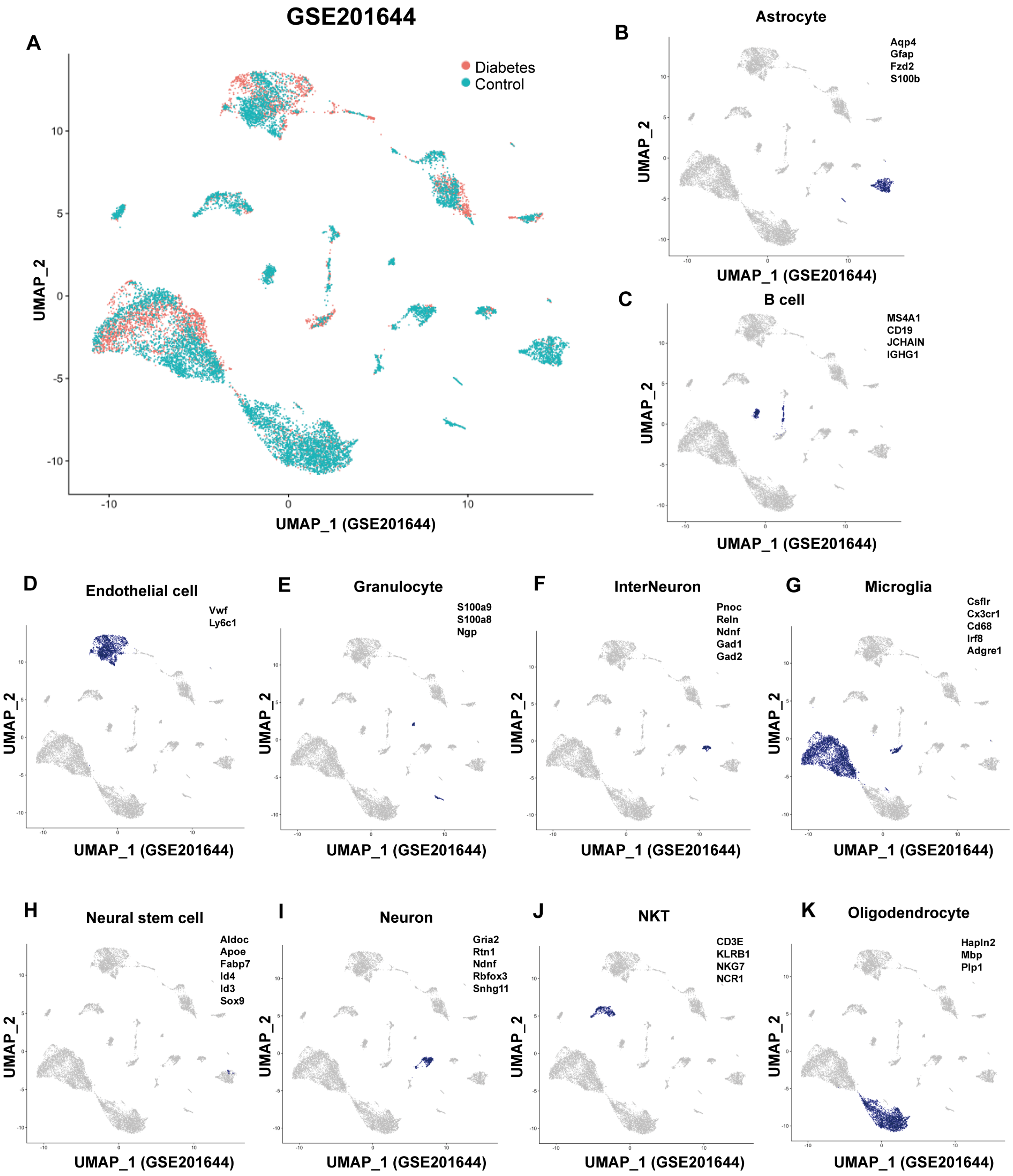

Supplemental Figure 4 Analysis of single-cell RNA sequencing (scRNA-seq) data of mouse hippocampal cells shows expression levels of lineage marker genes on a uniform manifold approximation and projection (UMAP) plots of all cells (Related to Figure 5). Visualization of the hippocampal cell clusters of a published scRNAseq data (GSE201644) was performed on a 2D map with UMAP. **A**, showed UMAP plot of all cells acquired by scRNA-seq of mouse hippocampal samples. Cells were obtained from mouse hippocampus of control group (blue) and the db/db

diabetes group (red). **B-K**, Expression levels of lineage marker genes of astrocyte (**B**), B cell (**C**), endothelial cell (**D**), granulocyte (**E**), interneuron (**F**), microglia (**G**), neural stem cell (**H**), neuron (**I**), NKT (**J**), and oligodendrocyte (**K**) were shown on UMAP plots.

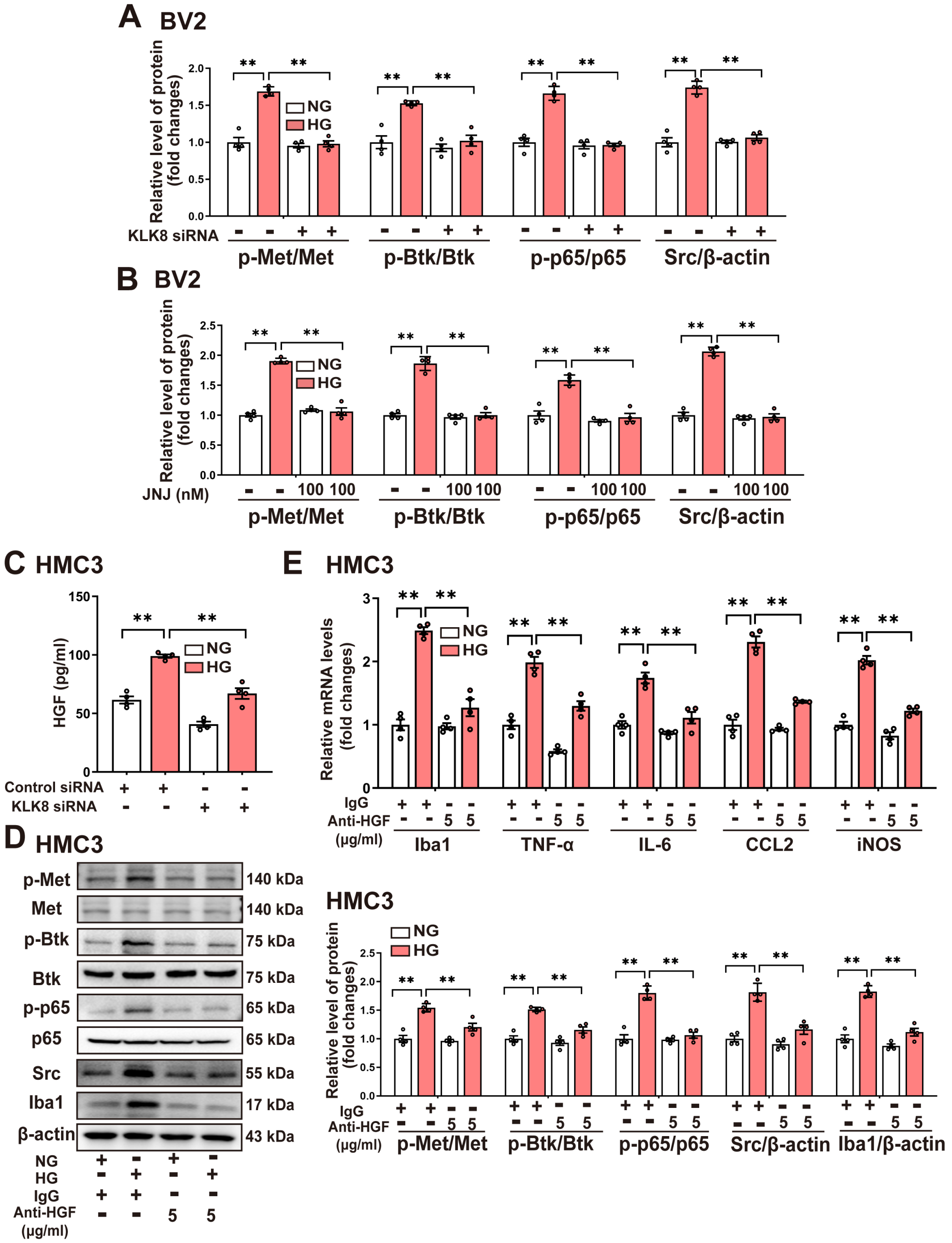

Supplemental Figure 5 High glucose enhances the release of HGF, thereby stimulating Met signaling and microglial activation via a mechanism dependent on KLK8 (Related to Figure 4). A, BV2 cells were transfected with control siRNA or KLK8 siRNAs, and then treated with NG or HG for 48 h. **B**, BV2 cells were treated with NG or HG with or without JNJ-38877605 at the indicated concentrations for 48 h. Relative densitometry of the p-Met/Met, p-Btk/Btk, p-p65/p65, Src/β-actin and Iba1/β-actin protein band in BV2 cells were shown in bar graphs(A, p-Met/Met: $F_{3,12} = 66.163$, $p < 0.001$. p-Btk/Btk: $F_{3,12} = 19.691$, $p < 0.001$. p-p65/p65: $F_{3,12} = 62.223$, $p < 0.001$. Src/β-actin: $F_{3,12} = 69.204$, $p < 0.001$; B, p-Met/Met: $F_{3,12} = 133.34$, $p < 0.001$. p-Btk/Btk: $F_{3,12} = 110.093$, $p < 0.001$. p-p65/p65: $F_{3,12} = 35.223$, $p < 0.001$. Src/β-actin: $F_{3,12} = 170.945$, $p < 0.001$)**.** **C,** HMC3 cells were transfected with control siRNA or KLK8 siRNAs, and then treated with NG or HG for 48 h. HGF contents in the cell medium were measured by ELISA assay ($F_{3,12} = 61.472$, $p < 0.001$). **D-E**, HMC3 cells were treated with NG or HG in the presence or absence of a fully human

anti-HGF neutralizing antibody Rilotumumab at the indicated concentrations for 48 h. **D**, Protein levels of p-Met, Met, p-Btk, Btk, p-p65, p65, Src, and Iba1 were determined by western blot analysis. Representative protein bands were presented on the left of the histograms (p-Met/Met: $F_{3,12} = 28.968$, $p < 0.001$. p-Btk/Btk: $F_{3,12} = 33.858$, $p < 0.001$. p-p65/p65: $F_{3,12} = 52.165$, $p < 0.001$. Src/β-actin: $F_{3,12} = 38.021$, $p < 0.001$. Iba1/β-actin: $F_{3,12} = 57.527$, $p < 0.001$). **E,** The mRNA levels of Iba1, TNF-α, IL-6, CCL2, and iNOS were detected by qRT-PCR (Iba1: $F_{3,12} = 66.043$, $p < 0.001$. TNF-α: $F_{3,12} = 72.577$, $p < 0.001$. IL-6: $F_{3,12} = 30.211$, $p < 0.001$. CCL2: $F_{3,12} = 106.547$, $p < 0.001$. iNOS: $F_{3,12} = 99.591$, $p < 0.001$). Data were presented as means ± SEM (n = 4, one-way ANOVA). ** $p < 0.01$. JNJ represents JNJ-38877605.

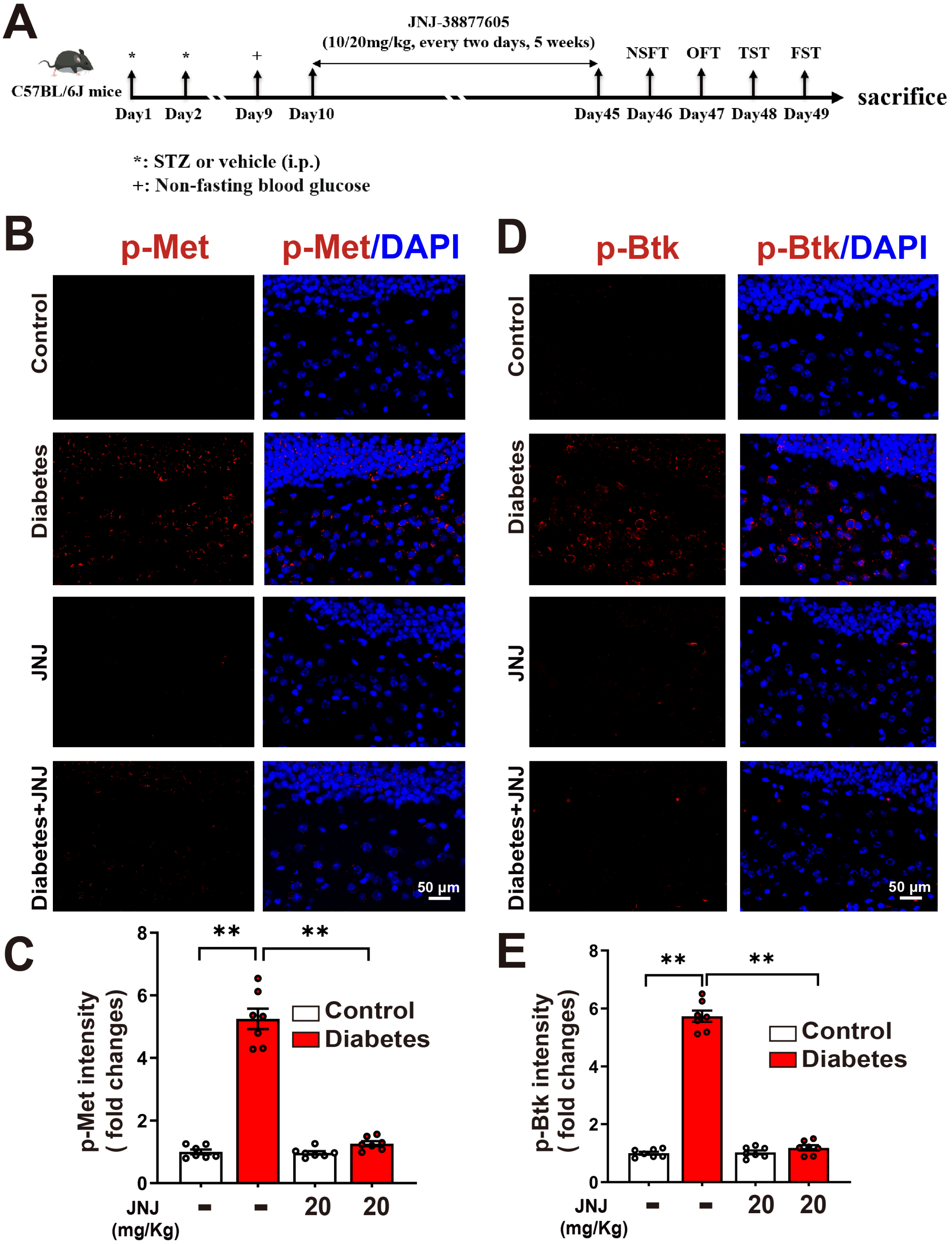

Supplemental Figure 6 Met inhibitor inactivates Met/Btk signaling pathway in the

hippocampus of STZ-induced diabetic mice (Related to Figure 5). Control or STZ-induced diabetic mice were intraperitoneally injected with the Met inhibitor JNJ-38877605 at the indicated concentrations once every two days for a period of 5 weeks. **A**, Timeline of the experimental procedures. **B**, Hippocampal sections were stained with fluorophore-labeled antibodies against phosphorylated Met (p-Met, red). DAPI staining was used to detect nuclei (blue). Scale bar = 50 μm. **C**, The quantification of the fluorescence intensity of p-Met ($F_{2,36\ (\text{Met inhibitor})} = 131.805$, $p < 0.001$; $F_{1,36\ (\text{treatment})} = 168.954$, $p < 0.001$; $F_{2,36\ (\text{Met inhibitor} \times \text{treatment})} = 127.827$, $p < 0.001$). **D**, Hippocampal sections were stained with fluorophore-labeled antibodies against phosphorylated Btk (p-Btk, red). DAPI staining was used to detect nuclei (blue). Scale bar = 50 μm. **E**, The quantification of the fluorescence intensity of p-Btk ($F_{2,36\ (\text{Met inhibitor})} = 387.05$, $p < 0.001$; $F_{1,36\ (\text{treatment})} = 452.692$, $p < 0.001$; $F_{2,36\ (\text{Met inhibitor} \times \text{treatment})} = 397.899$, $p < 0.001$). Data were presented as means ± SEM (n = 7, two-way ANOVA). ** $p < 0.01$. JNJ represents JNJ-38877605.

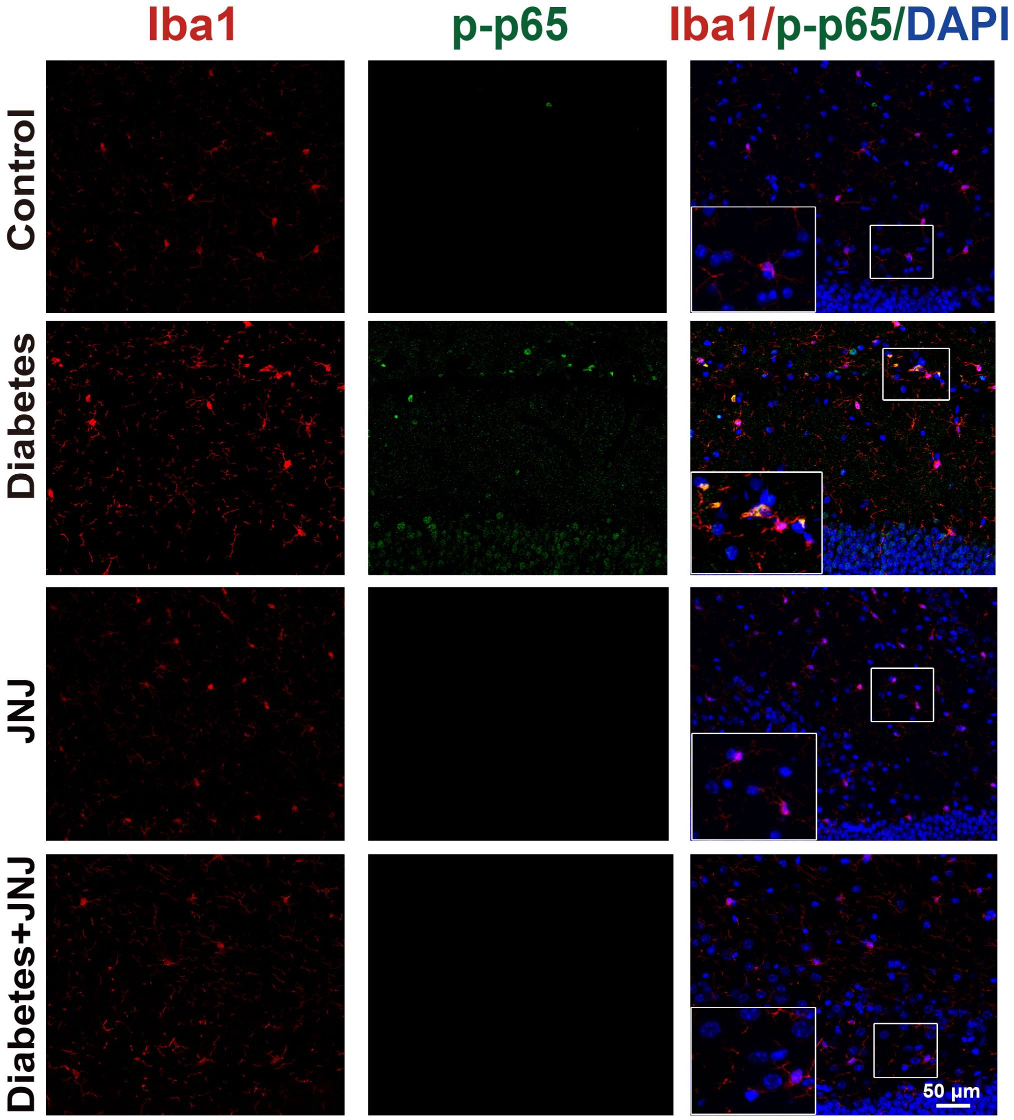

Supplemental Figure 7 Met inhibitor inactivates NF-κB in microglial cells in the hippocampus of STZ-induced diabetic mice (Related to Figure 5). Control or STZ-induced diabetic mice were intraperitoneally injected with the Met inhibitor JNJ-38877605 at the indicated concentrations once every two days for a period of 5 weeks**.** Hippocampal sections were stained with fluorophore-labeled antibodies against the microglial cell marker Iba1 (red) and phosphorylated p65 (p-p65, green). DAPI staining was used to detect nuclei (blue). The merge image represents double positive staining for Iba1 and p-p65. Areas in white boxes were shown enlarged. Scale bar = 50 μm.

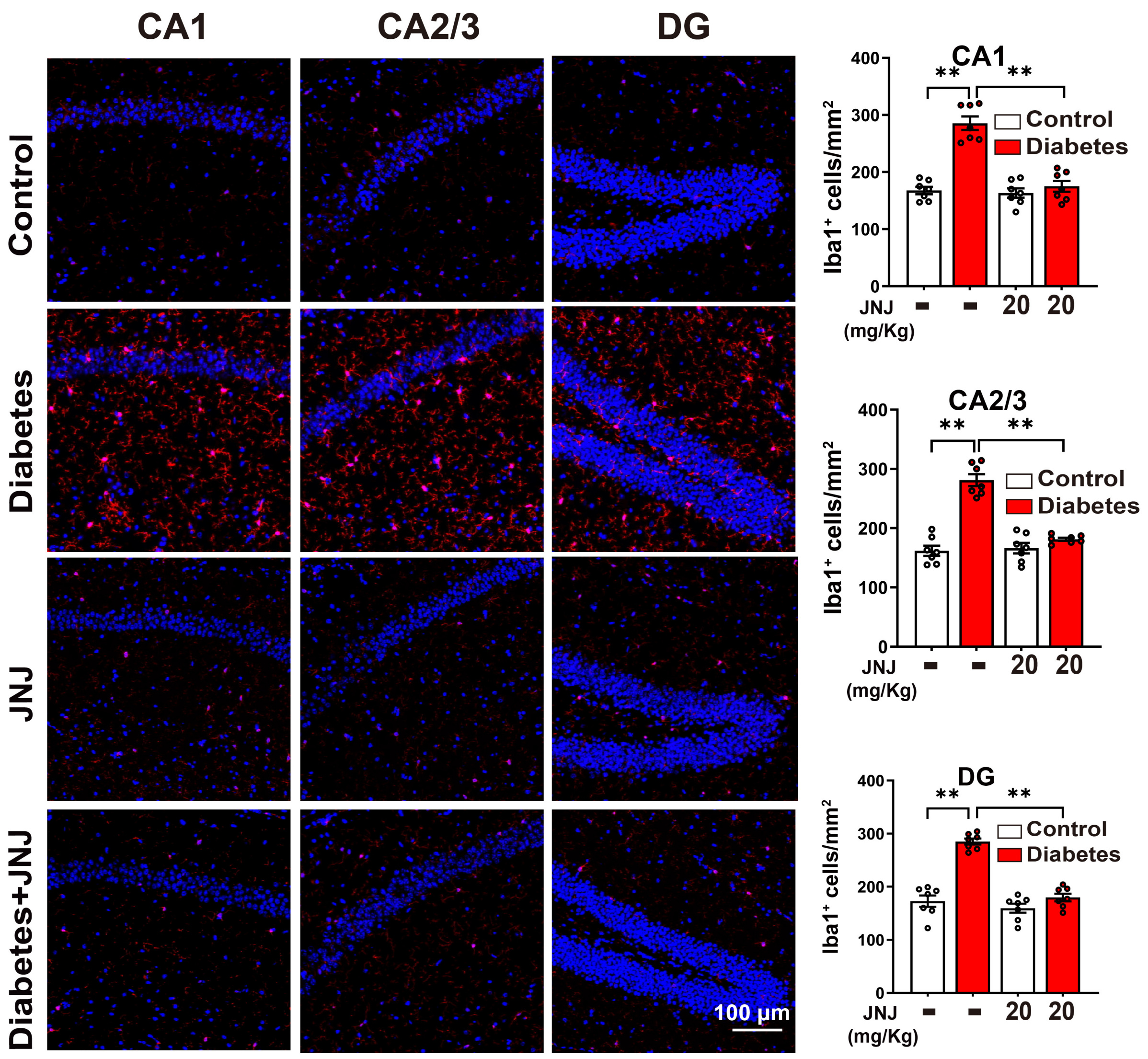

Supplemental Figure 8 Met inhibitor attenuates microglia activation in STZ-induced diabetic mice (Related to Figure 5). Control or STZ-induced diabetic mice were intraperitoneally injected with the Met inhibitor JNJ-38877605 at the indicated concentrations once every two days for a period of 5 weeks**.** Immunofluorescent staining showed Iba1 (red) expression in the CA1, CA2/3, and DG subregions of the hippocampus. Nuclei were counterstained with DAPI (blue). Scale bar = 100 μm (CA1: $F_{1,24}$ (Met inhibitor) = 38.906, $p < 0.001$; $F_{1,24}$ (treatment) = 49.391, $p < 0.001$; $F_{1,24}$ (Met inhibitor×treatment) = 33.144, $p < 0.001$. CA2/3: $F_{1,24}$ (Met inhibitor) = 35.341, $p < 0.001$; $F_{1,24}$ (treatment) = 69.981, $p < 0.001$; $F_{1,24}$ (Met inhibitor×treatment) = 42.205, $p < 0.001$. DG: $F_{1,24}$ (Met inhibitor) = 51.754, $p < 0.001$; $F_{1,24}$ (treatment) = 64.697, $p < 0.001$; $F_{1,24}$ (Met inhibitor×treatment) = 31.351, $p < 0.001$). Data were presented as means ± SEM (n = 7, two-way ANOVA). ** $p < 0.01$. JNJ represents JNJ-38877605.

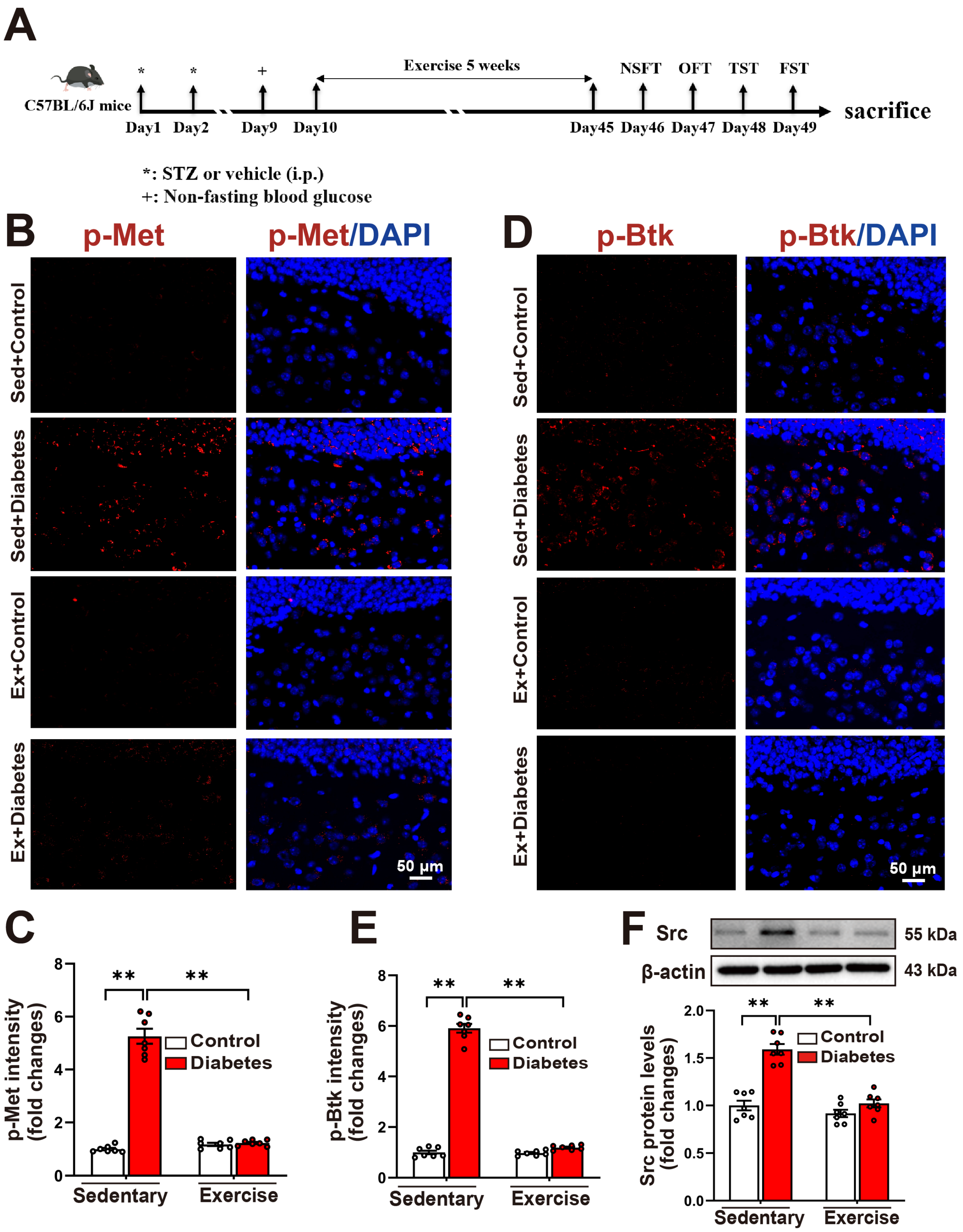

Supplemental Figure 9 Running exercise inactivates Met/Btk signaling pathway in the hippocampus of STZ-induced diabetic mice (Related to Figure 7). Control or STZ-induced diabetic mice were subjected to moderate intensity treadmill training for 5 weeks. **A**, Timeline of the experimental procedures. **B**, Hippocampal sections were stained with fluorophore-labeled antibodies against phosphorylated Met (p-Met, red). DAPI staining was used to detect nuclei (blue). Scale bar = 50 μm. **C**, The quantification of the fluorescence intensity of p-Met ($F_{1,24\ (\text{running exercise})} = 167.010$, $p < 0.001$; $F_{1,24\ (\text{treatment})} = 209.306$, $p < 0.001$; $F_{1,24\ (\text{running exercise}\times\text{treatment})} = 199.197$, $p < 0.001$). **D**,

Hippocampal sections were stained with fluorophore-labeled antibodies against phosphorylated Btk (p-Btk, red). DAPI staining was used to detect nuclei (blue). Scale bar = 50 µm. **E**, The quantification of the fluorescence intensity of p-Btk ($F_{1,24\ (running\ exercise)} = 583.424$, $p < 0.001$; $F_{1,24\ (treatment)} = 681.861$, $p < 0.001$; $F_{1,24\ (running\ exercise \times treatment)} = 571.613$, $p < 0.001$). **F**, Protein levels of Src in hippocampal tissues were examined by western blotting. Representative protein bands were presented on the top of the histograms. **F**, Protein levels of Src in hippocampal tissues were examined by western blotting. Representative protein bands were presented on the top of the histograms ($F_{1,24\ (running\ exercise)} = 47.003$, $p < 0.001$; $F_{1,24\ (treatment)} = 53.757$, $p < 0.001$; $F_{1,24\ (running\ exercise \times treatment)} = 26.071$, $p < 0.001$). Data were presented as means ± SEM (n = 7, two-way ANOVA). ** $p < 0.01$.

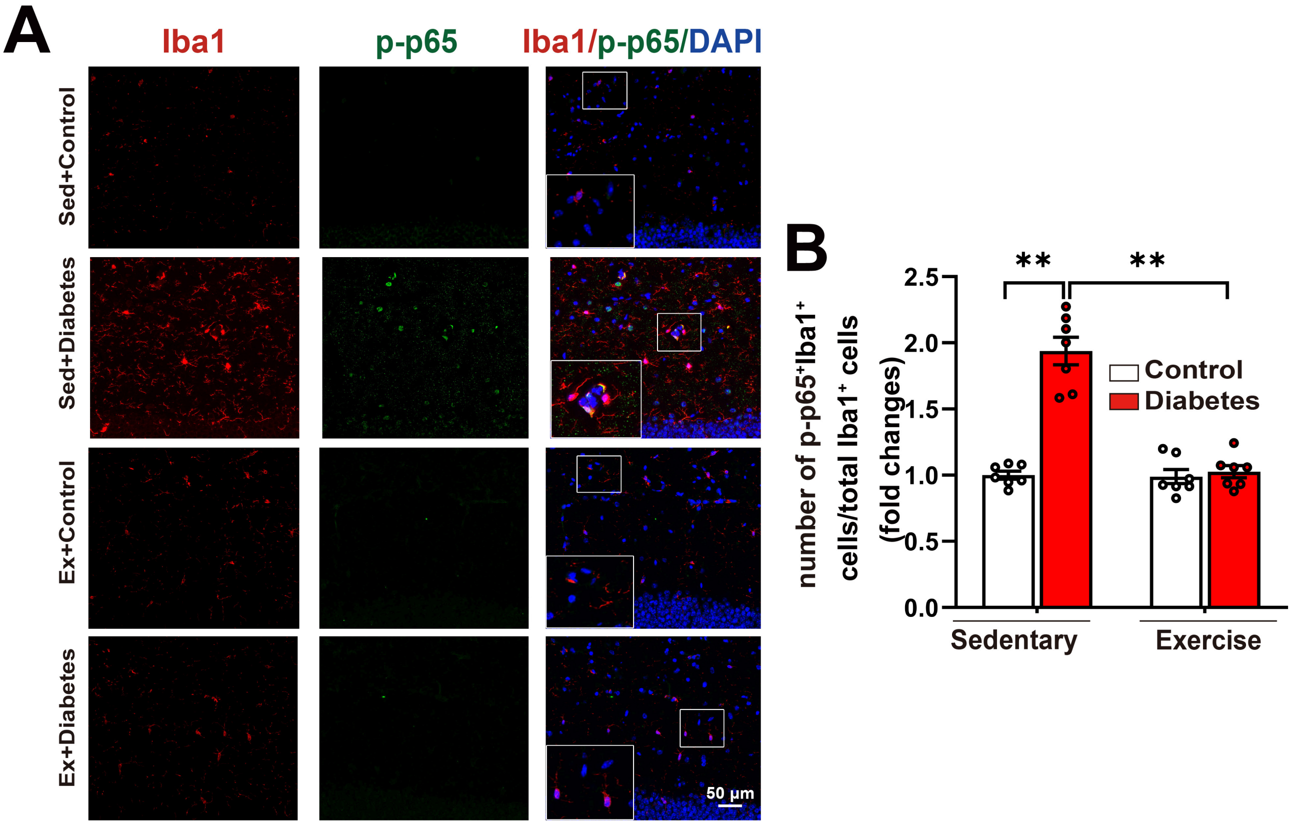

Supplemental Figure 10 Running exercise inactivates NF-κB in microglial cells in the hippocampus of STZ-induced diabetic mice (Related to Figure 7). Control or STZ-induced diabetic mice were subjected to moderate intensity treadmill training for 5 weeks. A, Hippocampal sections were stained with fluorophore-labeled antibodies against the microglial cell marker Iba1 (red) and phosphorylated p65 (p-p65, green). DAPI staining was used to detect nuclei (blue). The merge image represents double positive staining for Iba1 and p-p65. Areas in white boxes were shown enlarged. Scale bar = 50 µm. **B,** The quantification of the percentage of $Iba1^{+}$/$p\text{-}p65^{+}$ cells in total $Iba1^{+}$ cells ($F_{1,24\ (running\ exercise)} = 51$, $p < 0.001$; $F_{1,24\ (treatment)} = 56.93$, $p < 0.001$; $F_{1,24\ (running\ exercise \times treatment)} = 48.71$, $p < 0.001$). Data were presented as means ± SEM (n = 7, two-way ANOVA). ** $p < 0.01$.

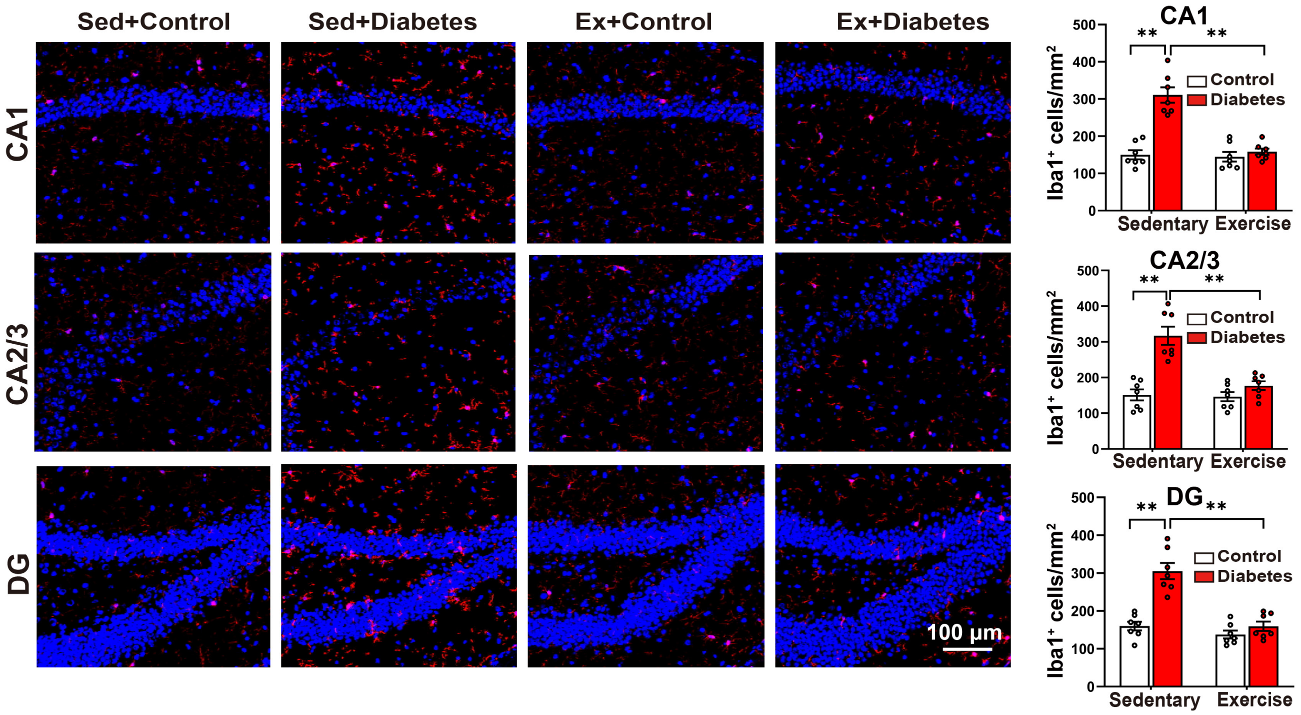

Supplemental Figure 11 Running exercise attenuates microglia activation in STZ-induced diabetic mice (Related to Figure 7). Control or STZ-induced diabetic mice were subjected to moderate intensity treadmill training for 5 weeks. Immunofluorescent staining showed Iba1 (red) expression in the CA1, CA2/3, and DG subregions of the hippocampus. Nuclei were counterstained with DAPI (blue). Scale bar = 100 μm. The quantification of $Iba1^+$ cell numbers in each subfield of the hippocampus were presented in the right panels (CA1: $F_{1,24\ (running\ exercise)} = 30.348$, $p < 0.001$; $F_{1,24\ (treatment)} = 37.189$, $p < 0.001$; $F_{1,24\ (running\ exercise×treatment)} = 26.515$, $p < 0.001$. CA2/3: $F_{1,24\ (running\ exercise)} = 17.621$, $p < 0.001$; $F_{1,24\ (treatment)} = 32.211$, $p < 0.001$; $F_{1,24\ (running\ exercise×treatment)} = 15.279$, $p < 0.001$. DG: $F_{1,24\ (running\ exercise)} = 33.171$, $p < 0.001$; $F_{1,24\ (treatment)} = 32.722$, $p < 0.001$; $F_{1,24\ (running\ exercise×treatment)} = 17.916$, $p < 0.001$). Data were presented as means ± SEM (n = 7, two-way ANOVA). ** $p < 0.01$.

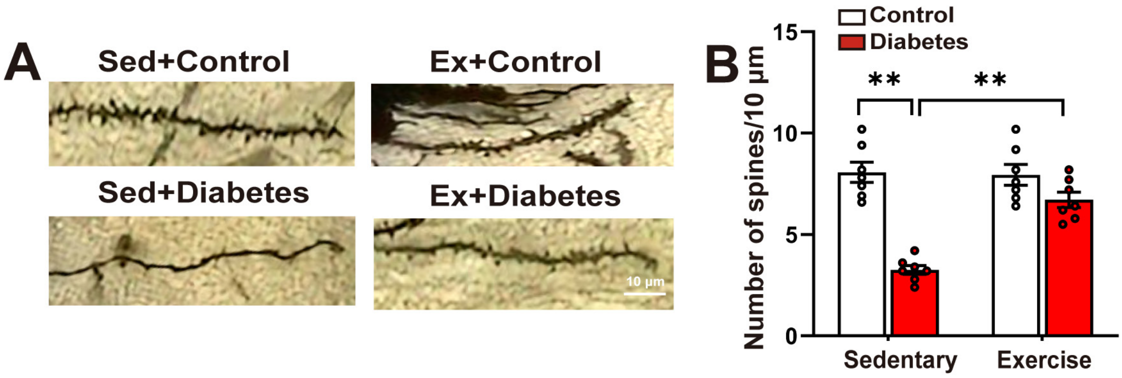

Supplemental Figure 12 Running exercise attenuates dendritic spine density in hippocampal neurons of STZ-induced diabetic mice (Related to Figure 8). Control or STZ-induced diabetic mice were subjected to moderate intensity treadmill training for 5 weeks. **A**, Representative microphotograph of Golgi-Cox staining in the hippocampal sections. Scale bar = 10 μm. **B**, Quantification of dendritic spine density of neurons in the hippocampus ($F_{1,24\ (running\ exercise)} = 15.754$, $p < 0.001$; $F_{1,24\ (treatment)} = 51.924$, $p < 0.001$; $F_{1,24\ (running\ exercise×treatment)} = 18.282$, $p < 0.001$). Data were presented as means ± SEM (n = 7, two-way ANOVA). ** $p < 0.01$.
